# Clinical outcomes of deep brain stimulation for obsessive‐compulsive disorder: Insight as a predictor of symptom changes

**DOI:** 10.1111/pcn.13619

**Published:** 2023-12-05

**Authors:** Nicola Acevedo, Susan Rossell, David Castle, Clare Groves, Mark Cook, Peter McNeill, James Olver, Denny Meyer, Thushara Perera, Peter Bosanac

**Affiliations:** ^1^ Centre for Mental Health Swinburne University of Technology Melbourne Victoria Australia; ^2^ St Vincent's Hospital Melbourne Victoria Australia; ^3^ Centre for Addiction and Mental Health University of Toronto Toronto Ontario Canada; ^4^ Clarity Health Melbourne Victoria Australia; ^5^ Department of Psychiatry University of Melbourne Melbourne Victoria Australia; ^6^ Bionics Institute East Melbourne Victoria Australia; ^7^ Department of Medical Bionics The University of Melbourne Melbourne Victoria Australia

**Keywords:** deep brain stimulation, electrode localization, insight, neurostimulation, obsessive‐compulsive disorder

## Abstract

**Aim:**

Deep brain stimulation (DBS) is a safe and effective treatment option for people with refractory obsessive‐compulsive disorder (OCD). Yet our understanding of predictors of response and prognostic factors remains rudimentary, and long‐term comprehensive follow‐ups are lacking. We aim to investigate the efficacy of DBS therapy for OCD patients, and predictors of clinical response.

**Methods:**

Eight OCD participants underwent DBS stimulation of the nucleus accumbens (NAc) in an open‐label longitudinal trial, duration of follow‐up varied between 9 months and 7 years. Post‐operative care involved comprehensive fine tuning of stimulation parameters and adjunct multidisciplinary therapy.

**Results:**

Six participants achieved clinical response (35% improvement in obsessions and compulsions on the Yale Brown Obsessive Compulsive Scale (YBOCS)) within 6–9 weeks, response was maintained at last follow up. On average, the YBOCS improved by 45% at last follow up. Mixed linear modeling elucidated directionality of symptom changes: insight into symptoms strongly predicted (*P* = 0.008) changes in symptom severity during DBS therapy, likely driven by initial changes in depression and anxiety. Precise localization of DBS leads demonstrated that responders most often had their leads (and active contacts) placed dorsal compared to non‐responders, relative to the Nac.

**Conclusion:**

The clinical efficacy of DBS for OCD is demonstrated, and mediators of changes in symptoms are proposed. The symptom improvements within this cohort should be seen within the context of the adjunct psychological and biopsychosocial care that implemented a shared decision‐making approach, with flexible iterative DBS programming. Further research should explore the utility of insight as a clinical correlate of response. The trial was prospectively registered with the ANZCTR (ACTRN12612001142820).

Obsessive‐compulsive disorder (OCD) is characterized by recurrent, intrusive, unwanted thoughts, images, or impulses [obsessions] and repetitive behaviors or mental acts in response to these [compulsions].^1^ OCD and related disorders are some of the costliest, functionally disabling and treatment resistant brain conditions.^2^ First line treatment of selective serotonin re‐uptake inhibitors (SSRIs) and cognitive behavioral therapy (CBT) lead to suboptimal outcomes; 40–60% remain treatment resistant and 60–80% relapse.^3^ Around 10% of OCD patients continue to experience highly disabling symptoms^4^ and may be eligible for deep brain stimulation (DBS).

The prevailing neurobiological model of OCD contends that hyperactivity and hyperconnectivity of cortico‐striato‐thalamo‐cortical (CSTC) circuits underlies OCD pathophysiology.^5^, ^6^ Deep brain stimulation (DBS) is an invasive neuromodulation therapy that provides a unique opportunity to modulate underlying CSTC circuitry in a personalized manner,^7^ leading to significant global and symptomatic changes in many otherwise refractory patients.

DBS for OCD is approved (under a humanitarian exception) by the US Food and Drug Administration (FDA) and Conformitè Europëenne (CE), and a 60% response rate is well‐established.^8^, ^9^ An estimated 440 people with OCD have been implanted worldwide across several randomized controlled trials, as such, meeting the World Society for Stereotactic and Functional Neurosurgery (WSFFN) criteria of an established therapy for *refractory* OCD.^9^, ^10^, ^11^ Yet, the level of evidence and efficacy has not translated into regulatory acceptance; many barriers to treatment access remain^12^ and the therapy remains investigational in nature.

The purpose of this trial was to investigate determinants of DBS efficacy in TR‐OCD. Existing literature suggests that certain symptom profiles influence response. Pre‐operative factors associated with good response include sexual/religious symptoms, older age of onset and insight into symptoms.^13^, ^14^ Hoarding and symmetry obsessions, comorbid personality disorder and greater depression are associated with poor response.^13^, ^15^, ^16^, ^17^ Yet, these clinical factors cannot predict response on an individual level, nor should they be used to deny individuals' therapy.

The precise location of stimulation relative to an anatomical target, or neuronal tract, influences clinical outcomes.^18^ Yet, a wide variety of regions are targeted (Fig. 1), with many reports failing to delineate the precise stimulation location. Further, our recent systematic review,^19^ identified that the extent and quality of follow up care is critical to patient outcomes, specifically (1) consistent and ongoing DBS programming, and (2) adjunct cognitive behavioral therapy (CBT); but few international sites have examined the utility of these factors.^16^, ^20^, ^21^, ^22^, ^23^

**Fig. 1:**
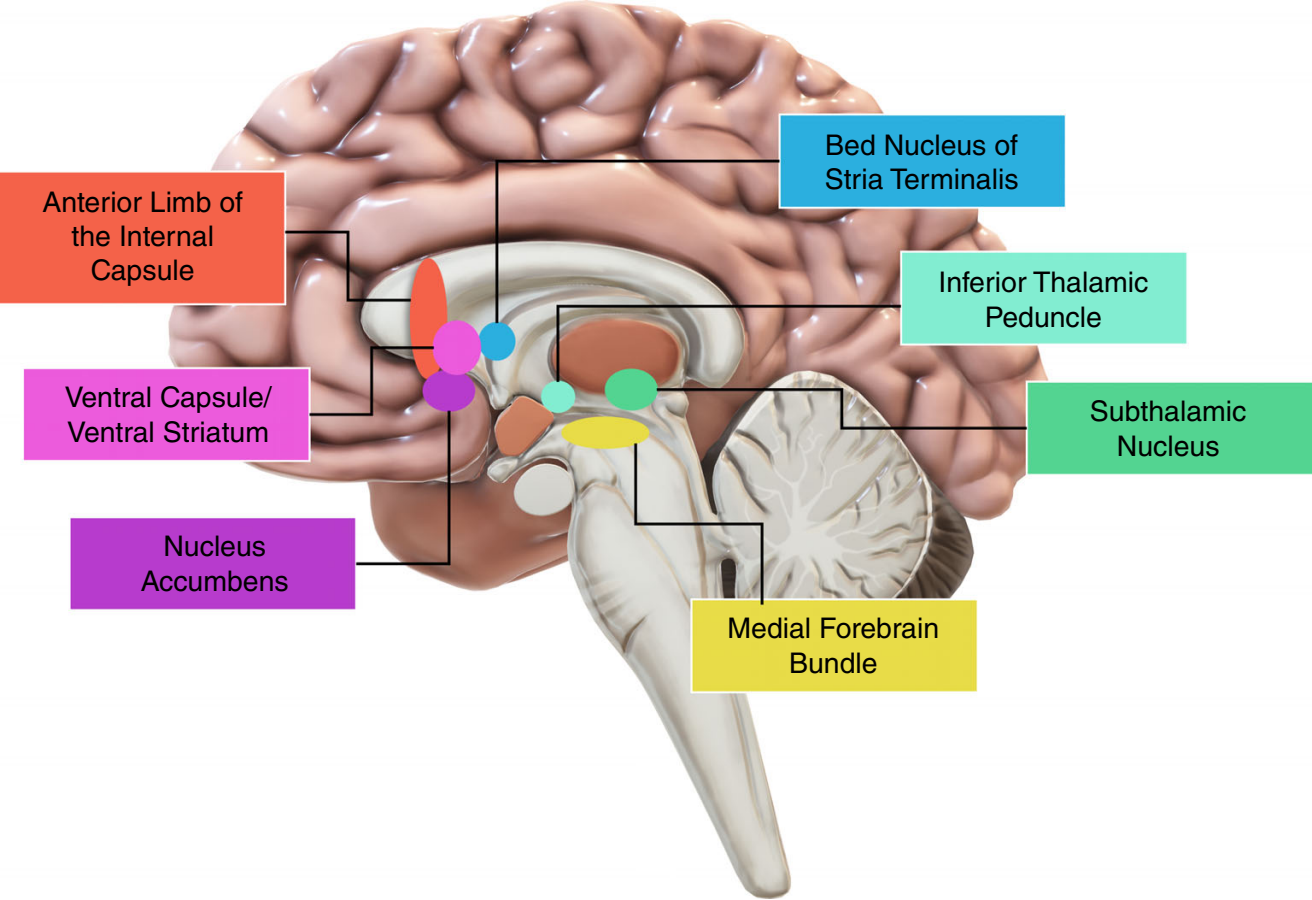
DBS targets for OCD the anterior limb of the internal capsule, the ventral capsule/ventral striatum, nucleus Accumbens, bed nucleus of stria terminalis, inferior thalamic peduncle, subthalamic nucleus, and the medial forebrain bundle.

There is thus a pressing need to better understand which patients and which treatment protocols are most likely to be associated with clinical benefit for people with OCD undergoing DBS. In this context, the current study implemented a comprehensive treatment protocol to optimize DBS therapy for a cohort of people with TR‐OCD.

The aims of the study were to: (1) assess the clinical efficacy of DBS therapy for TR‐OCD, including primary and comorbid symptoms; (2) investigate predictors of OCD symptom improvement in response to DBS therapy (both clinical characteristics and lead placement were explored); and (3) report safety data.

## Methods

### Participants

Eight participants (three females) were recruited *via* referral from their treating psychiatrist to the principal investigator (P.B.), at St Vincent's Hospital Melbourne, Australia, between July 2012 and August 2021, and received approval by the Mental Health Tribunal, Victoria. Inclusion criteria included a Diagnostic and Statistical Manual of Mental Disorders (DSM‐IV) diagnosis of OCD, aged between 18 and 65 years, YBOCS ≥24, at least a 5‐year history of OCD with substantial functional impairment on DSM‐IV criterion C, global assessment of functioning (GAF) <45, and treatment refractoriness (≥2 SSRIs at maximum dosage for 12 weeks, one treatment with clomipramine hydrochloride at maximum dosage for at least 12 weeks, one augmentation trial with a second generation antipsychotic for 8 weeks in combination with a SSRI, and ≥16 CBT sessions). Exclusion criteria included a clinically significant comorbid DSM‐IV diagnosis (except for major depression disorder [MDD], and mild anxiety disorders), and clinically significant and unstable neurological or medical illness. The project was approved by St Vincent's Hospital Melbourne Human Research Ethics Committee (HREC/12SVHM/64) and conformed to the provisions of the Declaration of Helsinki. All participants provided written informed consent.

### Surgical procedure

**Fig. 2:**
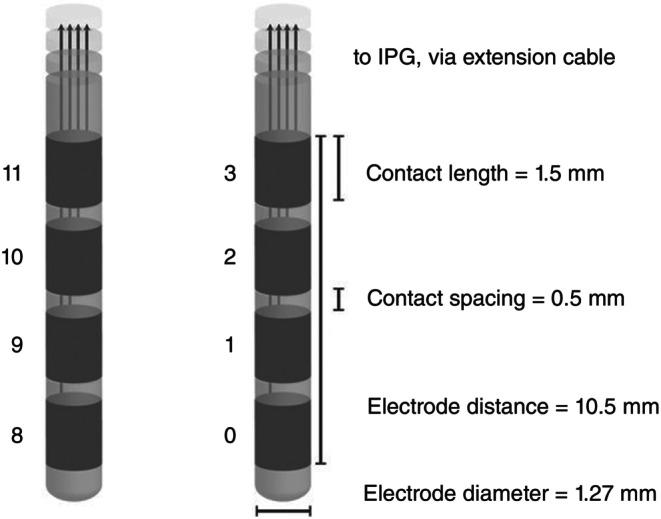
Medtronic 3389 leads: four contacts 1.5 mm long, spaced 0.5 mm apart.

Implantation of DBS leads was performed according to standard stereotactic procedures using frame‐based magnetic resonance imaging (MRI) for target determination, under local anesthesia. Participants underwent bilateral implantation of quadripolar 3389 electrodes (Medtronic, Minnesota, USA), spaced 0.5 mm apart and 1.5 mm long (Fig. 2). The surgical target (of the electrode tip) was the NAc as identified on MRI. Stereotactic atlases place this 7 mm lateral to the midline, 3 mm anterior to the anterior border of the anterior commissure, and 4 mm inferior to the intercommissural line. The two ventral contacts were targeted in the center of the NAc (E0, E1, E8, E9). Intra‐operative test stimulation was conducted to assess mood and behavior. The electrodes were connected *via* subcutaneous extensions to Activa PC, or RC stimulator (Medtronic, Minnesota, USA) placed in the infraclavicular pocket, under general anesthesia. Post‐operative computed tomography (CT) fused with pre‐operative MRI verified the position of the implanted electrodes. DBS was switched on 2 days following the surgery.

### Programming

Anatomical guidance (fused imaging) was used to select the active electrode for initial programming. Programming was conducted in accordance with the Montgomery algorithm^24^; initial parameters were 130 Hz, 90 μs, voltage was increased by 0.5 V increments, until sufficient benefit or a limiting side effect was reached (see Figure S1.). The programming adjustments involved a shared decision‐making process between the psychiatrist (P.B.) and participant; benefit was defined by a global assessment of subjective reports and clinical impression.

### Study design

1. Optimization phase: during the first 8‐months of therapy, stimulation parameters were assessed and adjusted fortnightly. Once a 6‐point drop in YBOCS was obtained, fortnightly CBT sessions commenced, and maintained for 24‐weeks. CBT sessions focused on exposure and response prevention (ERP) as well as dealing with habits that accrued during long duration of OCD symptoms. Medications were maintained during this phase.
2. Maintenance phase: programming assessments were conducted every 3 months, and thereafter reduced in frequency depending on the participants' needs. Medication was reduced where possible. Outreach psychosocial support was provided when appropriate (unavailable for participants 7 and 8) by a senior mental health clinician weekly or fortnightly for 12 months, and monthly thereafter. The outreach therapy employed an eclectic approach, drawing from various treatment models including exposure response prevention, dialectic behavioral therapy, acceptance and commitment therapy, trauma informed care, and psychoeducation (see^25^ for further discussion).

A one‐month randomized controlled closed label phase was planned, but the first six participants declined due to fear of the therapy being terminated. Thus, the closed label condition was removed from the protocol.

### Outcome measures

Our primary outcome was the YBOCS to evaluate obsessive and compulsive symptoms, on a 40‐point scale.^26^ Clinical response was defined as a 35% improvement in the YBOCS total score from baseline. A range of secondary outcomes were collected. Depression was evaluated using the Hamilton Scale for Depression (HAMD).^27^ Anxiety was assessed using the Hamilton Anxiety Scale (HAMA).^28^ Insight into obsessive and compulsive symptoms was evaluated using the Brown Assessment of Beliefs Scale (BABS), a lower score indicating better insight.^29^ Additional neuropsychological, neuroimaging, functional and qualitative assessments were conducted and will be reported elsewhere.^30^, ^31^ The protocol was registered on a clinical trial registry (ACTRN12612001142820).

### Analyses

Clinical outcomes are reported using descriptive statistics as within‐ and between‐subject change from baseline. Mixed linear modeling was used to investigate predictors of change in symptom severity: repeated measure = weeks; random factor = participant; covariates/predictors = HAMA, HAMD, BABS; primary outcome variable = YBOCS (unless otherwise specified). The modeling considered each participant as a random effect and assigned a random intercept to model individual differences. The AR (1) covariance structure accounted for autocorrelation across repeated measures. Covariates were expressed as change variables (t1−(t1 − 1)). The first model included all timepoints within phase 1, which were approximately 2 weeks apart (termed ‘concurrent model’). The second model regressed the YBOCS change scores (after baseline) on the covariates from the previous assessment period (termed ‘lagged model’). The lagged model allowed determination of whether the value of covariates at a given time point could predict the next YBOCS score, approximately 2 weeks later (predicts Y(t) from X(t − 1)). Within time series analysis, residuals are likely autocorrelated, thus the outcome variable is also composed of a change variable to account for possible autocorrelation. The advantage of mixed linear modeling is that it is implemented for modeling case series data over time, allows for repeated or related measures, uneven datasets and missing data.

### Lead localization

DBS electrodes were localized using Lead‐DBS software (V2.5.3) (https://www.lead-dbs.org/).^32^ Briefly, pre‐operative T1 and T2‐weighted images and post‐operative computed topography (CT) scans were linearly coregistered using Advances Normalization Tools (ANTs) (http://stnava.github.io/ANTs/),^33^ and normalized into ICBM 2009b non‐linear Asymmetric (MNI) template space using the SyN approach implemented in ANTs. Coregistration and normalization were visually reviewed and refined if necessary. Some datasets required alternative coregistration and normalization processing methods. Subcortical refinement was applied as a module in Lead‐DBS to post‐operative acquisitions to correct for possible brain shift. DBS electrodes were then pre‐reconstructed using the PaCER algorithm^34^ and warped into MNI space. Lead reconstructions were modeled in a 3D subcortical atlas, the CIT‐168 Reinforcement Learning Atlas.^35^ Using Lead‐DBS Group, individual leads were reconstructed on a single MNI template to visualize the precise differences in surgical targeting of the NAc. The volume of tissue activated (VTA) for each lead was estimated using a finite element method^32^ based on the individual stimulation settings at last follow up, and visualized within MNI space using the CIT‐168 atlas.

## Results

### Descriptive statistics

**Fig. 3:**
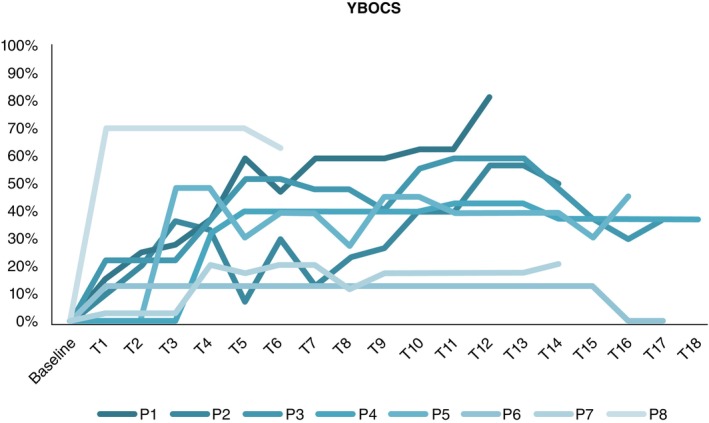
Individual percentage change in YBOCS. Programming adjustments and clinical assessments were conducted at approximately 2‐week intervals (T) during the optimization phase across approximately the first 9 months. The number of programming sessions during the optimization phase varied between participants based on individual needs.

**Fig. 4:**
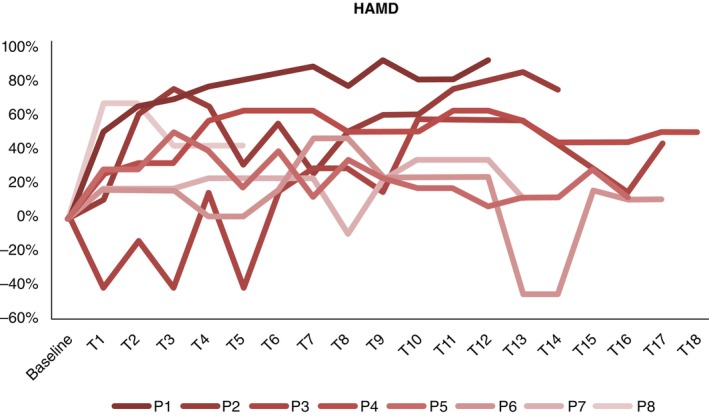
Individual percentage change in HAMD.

**Fig. 5:**
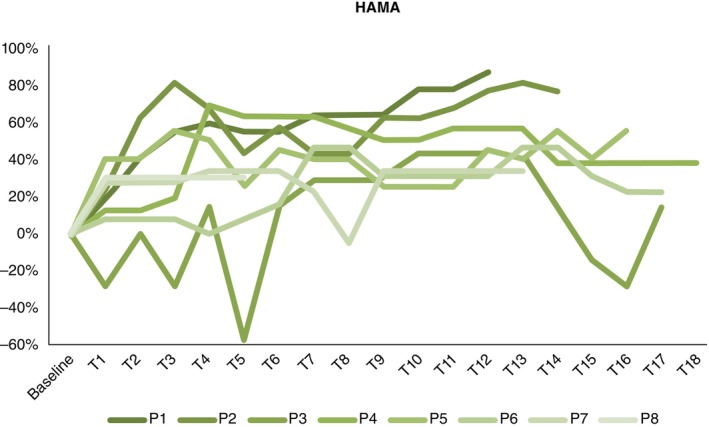
Individual percentage change in HAMA.

Table 1 provides baseline demographics and clinical characteristics of the participants. Figures 3, 4, 5 include individual patterns of response as a function of percentage change from baseline for YBOCS, HAMD and HAMA outcomes, within phase 1. Although clinical outcomes showed fluctuations within, and variability across participants, six individuals reached clinical response (75% response rate) for OCD symptoms; those who reached clinical response, did so within 6–9 weeks, and maintained response in the long term.

**Table 1:** Baseline demographics and clinical characteristics

ID	Age at OCD onset	Age	Gender	Main symptoms	Employment status	Relationship status	Level of education	Psychiatric comorbidities	Medications
1	10–14	29	Male	Checking, washing, ordering, previous trichotillomania.	Unemployed	Single	Tertiary (first year)	Severe anxiety, moderate depression, thoughts of self‐harm	Conazepam 20 mg, Duetiap 1500 mg, desven 500mg
2	12	36	Female	Contamination, checking, reassurance, avoidance.	Unemployed	Single	Secondary	Previous suicide attempt, bipolar, brain injury, chronic depression, post‐natal depression	Olanzapine 2.5 mg, Mirtazapine 30/45 mg, Fluoxetine 120 mg, Diazepam 10 mg
3	4	37	Female	Repetitive acts, self‐harm, excessive showering/washing, fear of imperfection.	Employed, student	Single	Tertiary	MDD, anxiety, admission for psychosis following excessive intake of thyroxine, Asperger's syndrome, possible PANDA	Escitalopram 80 mg, Aripiprazole 10 mg, Carbamazepine 200 mg, NAC 3 g
4	19	30	Male	Checking, tidying, ordering, fear of imperfection	Unemployed	Single	Secondary	Previous MDD, suicidal ideation, previous suicide attempt, previous hoarding behavior, previous psychiatric admissions	Risperidone 3 mg, Clomipramine 300 mg
5	10	51	Male	Washing, skin‐picking, sexual, paraphilia, guilt.	Unemployed	Single	Tertiary (withdrew)	PTSD following child sexual abuse, previous alcohol abuse, generalized anxiety, depression	Quetiapine 50 mg, Valprex 100 mg, Oxazpam 15 mg
6	18	32	Male	Checking, hoarding, contamination	Unemployed, student	Single	Tertiary (withdrew)	Previous severe depression, psychotic disorder, schizophrenia	Fluoxetine 100 mg, Quetiapine 100 mg, NAC 1 g
7	12	34	Male	Contamination, sexual, fear of imperfection/judgment, self‐injurious behavior, scrupulosity	Unemployed	Single	Secondary (withdrew) Tertiary (diploma)	Anxiety, history of epilepsy, possible narcissistic personality disorder	Quetiapine 100 mg, Clomipramine 125 mg
8	27	53	Female	Obsessional doubt, repetitive writing and affirmations	Unemployed	Single	Tertiary	Major depression, previous episode of bipolar disorder, previous episodes of hypomania	Olanzapine 7.5 mg, Quetiapine 200 mg, Lamotrigine 100 mg, Lithium 900 mg

**Fig. 6:**
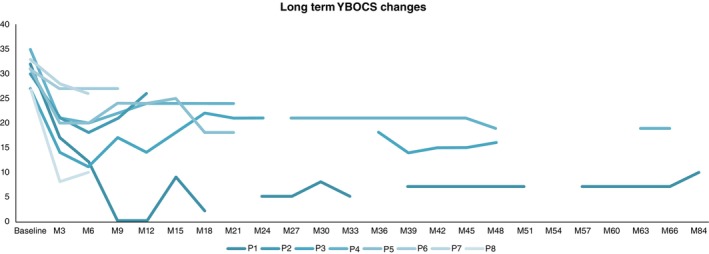
Individual changes in the YBOCS across the follow up period from baseline to 84 months (M).

Table 2 shows YBOCS change for each participant following phase 1 and last follow up, as well as final stimulation parameters. Figure 6 shows individual YBOCS outcomes at 3‐monthly intervals for the entirety of the follow up period. Last follow up varied from 9 months to 7 years, due to the time of implantation for each participant.

**Table 2:** Within subject YBOCS outcomes

Participant	Baseline YBOCS	Phase 1 improvement	Best response at	Improvement at last follow up	Last follow up	Final stimulation parameters
1	32	81%	1 year	69%	7 years	2– 3− C+; 10– 11− C+ 4.5 V, 130 Hz, 90 μs
2	30	50%	7 years	67%	7 years	2– 3− C+; 10– 11− C+ 4.5 V, 130 Hz, 90 μs
3	27	37%	1 year	41%	5.6 years	2– 3− C+; 10– 11− C+ 6.5 V, 150 Hz, 90 μs
4	35	37%	5 years	46%	6 years	2– 3− C+; 10– 11− C+ 7 V, 160 Hz, 90 μs
5	33	45%	2 months	45%	3.5 years	2– 3− C+; 10– 11− C+ 3 V, 130 Hz, 90 μs
6	31	13%	Non‐responder	13%	10 months	DBS explanted
7	33	21%	Non‐responder	18%	9 months	2– 3− C+; 10– 11− C+ 6.5 V, 130 Hz, 90 μs
8	27	63%	10 months	63%	10 months	0– 1− C+; 8– 9− C+ 3 V, 130 Hz, 90 μs

Stimulation parameters are shown as the active contact (−) and return contact (+).

Hz, Herts; μs, microsecond; V, volts.

Mean change in the clinical outcomes following phase 1 and last follow up are presented in Table 3; at last follow up mean change in YBOCS total score was 45%, YBOCS obsessions 44%, YBOCS compulsions 40%, HAMD 42%, HAMA 41%, and BABS 35%.

**Table 3:** Group level clinical outcomes

Outcome	Baseline (mean ± SD)	Phase 1	Mean change	Last follow up (mean ± SD)	Mean change
YBOCS total	30.7 ± 2.5	18 ± 6.8	41%	17 ± 6.4	45%
YBOCS obsessions	16.7 ± 4.6	8.6 ± 3.7	49%	9.3 ± 3.0	44%
YBOCS compulsions	14.6 ± 1.9	8.6 ± 2.6	41%	8.6 ± 3.6	40%
HAMD	16.25 ± 5.4	9.0 ± 5.1	44%	9.38 ± 3.5	42%
HAMA	15.9 ± 5.1	7.3 ± 2.6	54%	9.38 ± 2.3	41%
BABS	8.5 ± 3.3	5.3 ± 3.8	38%	5.50 ± 3.7	35%

The mean ± standard deviation is presented for each primary and secondary outcome. For patient 7 the last outcome for HAMD, HAMA, and BABS was carried forward.

Of the non‐responders, P6 showed elected to have the DBS device removed, while P7 experienced hypomanic symptoms from stimulation increases which interfered with and limited programming refinements.

### Mixed linear modeling

Residuals were tested for homoscedasticity and normality through visual inspection, which were met. A two‐tailed Spearman's correlation showed YBOCS raw scores were significantly correlated (*P* < 0.001) with HAMD (r = 0.68), HAMA (r = 0.59) and BABS (r = 0.56) raw scores. YBOCS change (t1−(t1 − 1)) scores were also significantly correlated (*P* < 0.001) with HAMD (r = 0.44), HAMA (r = 0.54), and BABS (r = 0.44) change scores.

Therefore, considering the associations between clinical variables with medium effect sizes, the relationship between clinical outcomes was further investigated using mixed linear modeling. The concurrent model analyzed predictors based on concurrent values (Table 4). Concurrent Model 1 showed that changes in HAMD and HAMA were significantly associated with the concurrent YBOCS change (*P* = 0.008, *P* = 0.013, respectively). Considering the previously established association with level of insight and clinical response^14^ we further investigated directionality by changing the outcome variable to the BABS. Model 2 showed that changes in HAMA significantly predicted concurrent BABS changes (*P* = 0.003).

**Table 4:** Models Statistics

Model	Predictors	Outcome variable	Coefficient (df)	t‐statistic	AR1 coefficient (SE)	*P*‐value
Concurrent predictor model						
1	HAMD Change**	YBOCS change	0.34 (98.30)	2.70	0.01 (0.24)	0.008
	HAMA Change*		0.38 (98.5)	2.51		0.013
	BABS Change		−0.06 (89.65)	−0.17		0.865
2	YBOCS Change	BABS change	−0.004 (98.70)	−0.17	0.15 (0.12)	0.860
	HAMD Change		0.06 (95.42)	1.92		0.051
	HAMA Change*		0.12 (98.61)	3.08		0.003
Lagged predictor model						
1	HAMD Change*	YBOCS change	0.37 (92.92)	2.60	−0.04 (0.16)	0.011
	HAMA Change		0.12 (92.82)	0.69		0.490
	BABS Change**		0.12 (92.82)	−2.71		0.008
2	YBOCS Change*	BABS change	−0.04 (84.89)	−2.06	−0.41 (0.14)	0.042
	HAMD Change*		0.06 (84.12)	2.05		0.043
	HAMA Change***		0.12 (86.23)	3.49		<0.001

Statistically significant predictors are bolded and denoted by * for *P*‐values <0.05, ** for *P*‐values <0.01, and *** for *P*‐values <0.001.

df, degrees of freedom; SE, standard error.

The lagged models further investigated directionality and lagged associations, by considering the previous change in covariates as predictors of subsequent (not current) YBOCS changes. Model 1 demonstrated that the previous HAMD and BABS were significantly associated with the ensuing YBOCS change (*P* = 0.011, *P*=0.008, respectively). Again, the outcome variable was changed to the BABS change variable. Model 2 demonstrated that the previous change in YBOCS, HAMD and HAMA were significantly associated with the consecutive BABS change (*P* = 0.042, *P* = 0.043, *P* = <0.001, respectively).

### Lead localization

**Fig. 7:**
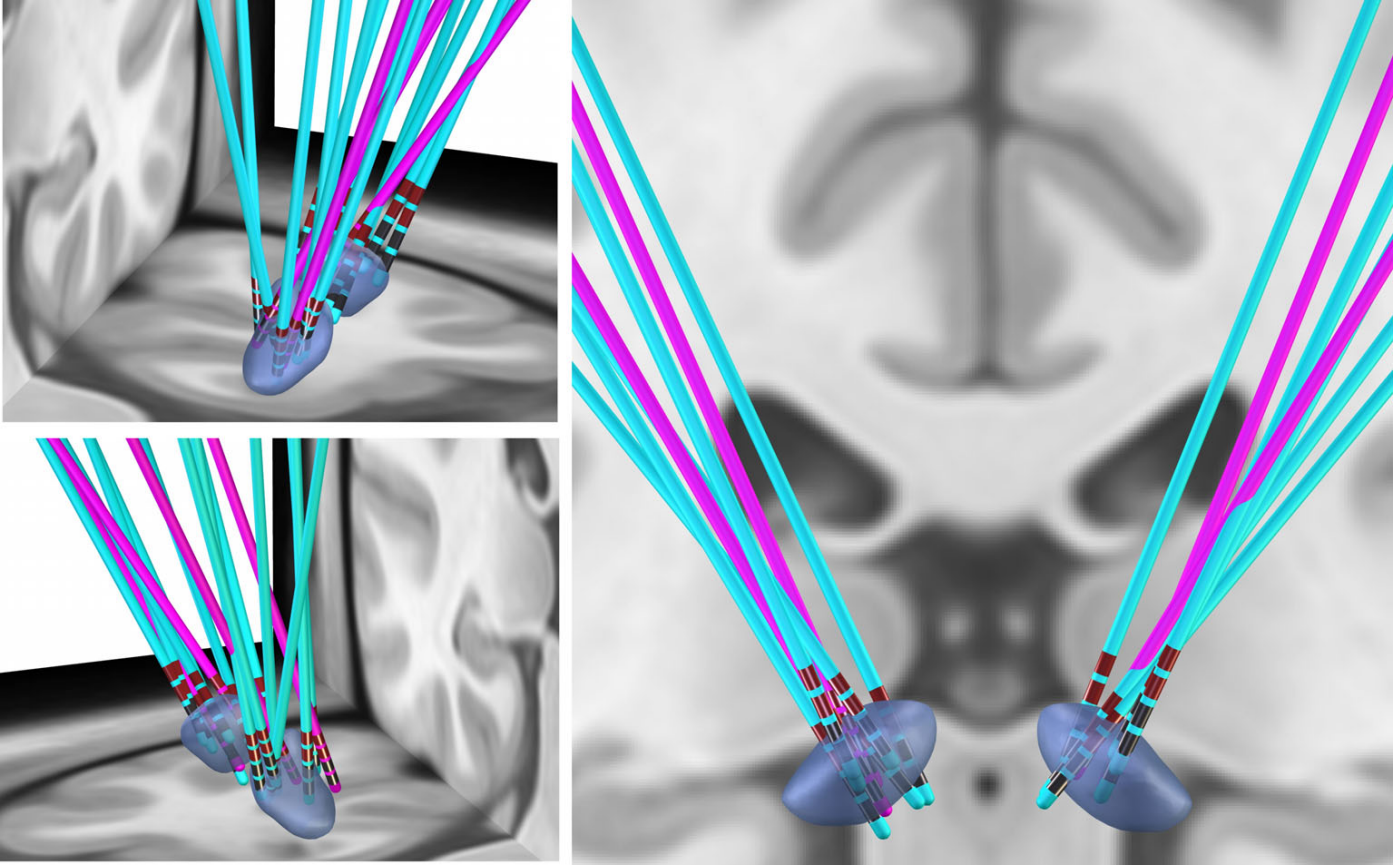
Individual patient leads are reconstructed on a normative atlas to demonstrate the slight differences in surgical placement within the NAc using the LeadDBS toolbox. DBS leads of responders are represented in blue, DBS leads of non‐responders are represented in purple.

DBS leads were reconstructed using the LeadDBS toolbox and visually evaluated. Group level visual inspection showed that five out of six responders had their leads placed more dorsal compared to non‐responders, relative to the NAc (Fig. 7). The chronic electrode contacts were the two most dorsal contacts for all participants, except for P8, who had experienced an insula hematoma and was receiving low level stimulation. Also, the responder's leads appear more posterior within the right hemisphere, yet this relationship is not observable in the left hemisphere. The localization of leads within 2D planes (Supporting Information, S2. and S3.), VTA estimates of final stimulation parameters (Supporting Information, S4), and 3D video of leads (Supporting Information, S5.) is provided in the Supporting Information to assist in the interpretation of leads relative to anatomical structures.

### Adverse events

Participant 3 had subcortical expressive dysphasia and generalized seizures, causing DBS to be switched off from 1 week following surgery for 6 weeks in tandem with neurological recovery. Participant 5 experienced labile affect and was admitted to hospital after 31 months of therapy when stimulation voltage was increased from 3V to 6V. The DBS surgery of participant 8 was complicated by a hematoma to the left insula and sylvian fissure, resulting in a transient lesion effect with expressive dysphasia; all neurological symptoms resolved over time and OCD remission was achieved. Participant 7 experienced fluctuating hypomania symptoms from 2 weeks of therapy, until last follow up, causing intermittent reduction in programming.

## Discussion

This study of eight people with severe treatment‐resistant OCD demonstrated the clinical efficacy of DBS, including patterns and predictors of symptom change. DBS therapy achieved a rapid, large, and sustained clinical effect. The mean change in YBOCS, the primary outcome, was 41% after the optimization phase (8 months), and 45% at last follow up (9 months–7 years). Clinical response was achieved in 75% of participants within 6–9 weeks and maintained in the long‐term. The HAMD, HAMA and BABS also showed clinically meaningful improvements. Mixed linear modeling demonstrated depression and insight as predictors of changes in symptom severity, yet insight showed a stronger statistical lagged effect across timepoints. Lead localization of DBS electrodes demonstrated that the majority of responders had their leads placed slightly more dorsal. Surgical and stimulation related side effects were experienced, consistent with previous reports.

Participants experienced improvement in primary symptoms, but also in various psychological functions. Primary (obsessions, compulsions) and secondary (anxiety, depression) symptoms, and symptomatic insight demonstrated statistical correlation. Changes in OCD symptoms showed some fluctuations, yet major improvements occurred within the first 6 months, and generally plateaued thereafter. However, anxiety and depression symptoms showed dramatic fluctuations within and across participants; two patients showed deterioration beyond baseline, which later improved and two other participants improved by over 80%‐ a greater extent to OCD symptom changes. These findings highlight the importance of considering several outcomes of DBS efficacy. This patient group exhibits complex phenotypes and etiologies; thus, it is necessary to consider various determinants of recovery in a functionally relevant manner. We recently reported various psychosocial changes, high satisfaction, and valuable feedback relating to DBS care within this cohort.^30^ Further, our lived experience investigation of responders and carers identified profound changes to psychopathology and self‐constructs; phenomenological changes are discussed within a proposed cognitive model.^31^

**Fig. 8:**
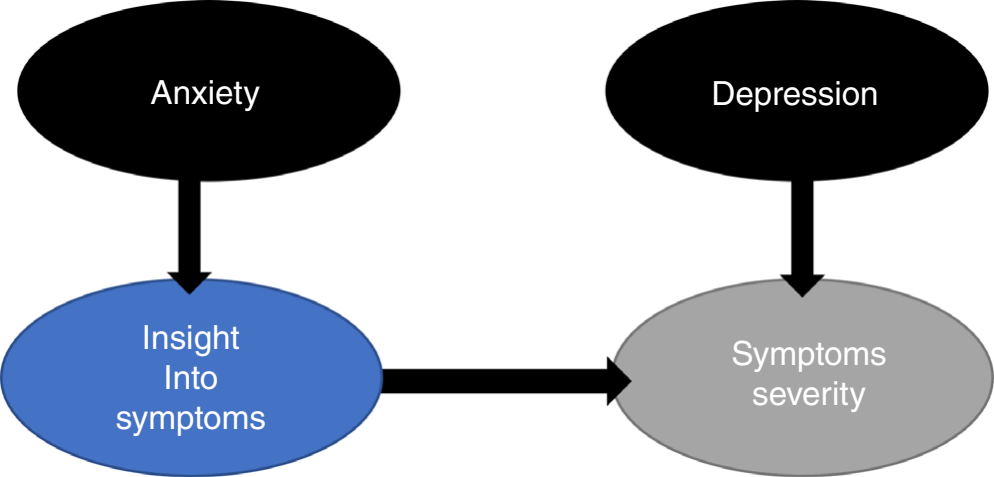
Schematic of mixed linear modeling outcomes representing directionality of symptom changes.

Changes in insight predicted subsequent symptom severity changes (*P* = 0.008). Yet, the initial symptomatic changes appeared to be driven by depression and anxiety that showed a concurrent effect on symptom severity. Previously, it has been proposed that DBS modulates anxiety within seconds to minutes, and obsessions within weeks,^36^ our analysis supports and add to this notion on the time‐series effect of DBS on various symptom domains. When considering the strongest predictors in each model, the analysis suggests that changes in depression and anxiety are occurring concurrently and driving subsequent changes in symptom severity and insight. More specifically, depression had a stronger effect on symptom severity, and anxiety had a stronger effect on insight across time points (lagged effect). In turn, the level of insight into symptoms is influencing subsequent changes in symptom severity (see Fig. 8 for a schematic). The analyses employed change variables, which eliminate autocorrelation that may still be present in raw scores and are more suggestive of causality.

The impact of comorbid anxiety and depression is well understood within OCD, yet the role of insight in relation to psychopathology and treatment response is underappreciated. Insight related to mental illness is a multidimensional, dynamic, and continuous phenomenon.^37^ Insight in OCD involves the understanding that one's obsessions and compulsions are irrational. Poor insight is present in around a third of people with OCD and is associated with greater illness severity, poorer prognosis, higher rates of comorbidities, and worse treatment response.^38^, ^39^, ^40^, ^41^, ^42^, ^43^ Improved insight of mental health can enhance treatment adherence and coping strategies.^41^ Therefore, insight is an important determinant of OCD psychopathology and prognosis. A recent large cohort analysis demonstrated good insight at baseline to have a positive predictive value of 84.4% for clinical response from DBS for OCD, yet level of insight did not yield individual predictive power.^14^ Programming is more difficult in those who lack awareness into cognitive‐affective functions, as the utility of subjective observations is limited.^44^ Insight is also related to symptom domains; individuals with perfectionism, hoarding or symmetry symptoms describe their symptoms as ego‐syntonic and do not respond as well as others.^16^ Our sample size does not allow for patient specific predictive analysis, yet it is worth noting that the non‐responders in our cohort had relatively poor baseline insight (BABS score of 11 and 14) although one of the responders had a comparable baseline score (BABS score of 14). Therefore, insight at baseline may serve as a useful prognostic, but not, exclusion factor, and needs consideration with other patient characteristics.

In previous studies, long‐term DBS stimulation of the NAc has led to 12–33% improvement in OCD symptoms, and response in 10–56%,^21^, ^45^, ^46^, ^47^, ^48^, ^49^, ^50^, ^51^, ^52^, ^53^ yet other targets (ventral anterior limb of the internal capsule (ALIC), ventral capsule/ventral striatum (VC/VS)) have been associated with up to 80% response.^19^ The higher efficacy of NAc DBS identified in the current study might be due to the detailed and comprehensive fine tuning of stimulation as well as the adjunct behavioral and psychosocial therapy.

We implemented a multi‐disciplinary model to support the OCD DBS treatment during the pre‐operative and post‐implantation periods. Psychiatric care involved extensive programming sessions with collaborative involvement from the participant, as well as exposure therapy, and continuing psychological support. Also, outreach psychosocial therapy with participants and families consolidated exposure therapy in naturalistic settings. Although DBS alone can achieve an initial and rapid improvement in OCD symptoms, it is contended that integrative therapy with a holistic consideration of the severity and impact of OCD symptoms on everyday life is necessary for sustained and optimized recovery. For further discussion, refer to our clinical guideline for managing people receiving DBS for TR‐OCD.^25^ Adjunct therapeutic approaches in this patient group are rarely discussed in the scientific literature.^54^ Yet, post‐operative management of psychiatric patients relies on a highly experienced and specialized multidisciplinary team working in collaboration with the patients and their families. Greater discussion in the scientific literature is required in this domain, with the aim to develop more standardized and personalized care.

The efficacy within our cohort, is also attributable to probable stimulation of regions dorsal to the surgical target. Five out of six responders in our study were receiving chronic stimulation in the two most dorsal contacts on the lead, which were placed above the NAc (Fig. 7) and likely modulating nearby structures, including the ventral ALIC. The ventral ALIC has been consistently identified as the optimized target across different cohorts^19^, ^20^, ^55^, ^56^, ^57^, ^58^ and we add to this growing body of literature. More precisely, localization of DBS leads has identified that stimulation of a tract transversing the ALIC and connecting areas of the prefrontal cortex to the subthalamic nucleus and mediodorsal nucleus of the thalamus has the greatest benefits.^18^, ^22^, ^45^, ^59^, ^60^, ^61^, ^62^ Despite modern surgical techniques, the placement of the leads cannot be assumed to be uniform across individuals. Thus, evaluation and reporting of clinically effective DBS contacts is critical to progress scientific understanding of the best practices to optimize efficacy. Therefore, we present the precise localization of DBS leads for transparency and greater understanding in the field.

Participants declined the planned closed label phase, in which they would have known that their stimulation may or may not be turned off. There are several concerns with DBS sham conditions in psychiatry. Turning DBS off can lead to drastic deterioration of symptoms^63^, ^64^; as a result, sham conditions are often terminated early.^55^, ^65^, ^66^ Also, order effects may occur^53^ and increased anxiety during blinded phases can complicate clinical evaluations. DBS efficacy relies on the optimization of programming, which can take 6–12 months, yet for effective blinding the participant should be naïve to stimulation; both cannot hold true in closed label conditions. As DBS is considered a ‘last resort’ treatment for extremely vulnerable patients, ethically we need to provide the best possible treatment options. Thus, sham methods were necessary in earlier investigations to establish a level of efficacy. However, we contend that a sufficient level of evidence has been reached to consider DBS an established therapy for TR‐OCD,^9^, ^10^, ^11^, ^67^, ^68^ and that closed label conditions do not satisfy ethical or validity requirements. The overall goal of DBS therapy is to improve outcomes for patients. Therefore, if the patient experiences clinically significant long‐term improvement, hasn't the endpoint been reached?

There are several limitations to this project, including the small number of participants, which limits extrapolation of findings. Having said that, we conducted the analyses with sample size at the forefront of our considerations, and the overall effect sizes were robust to analyses. There are a limited number of cases of DBS for OCD worldwide, and most are confined to single research groups, hence our cases expand the overall reported cohort and provide a perspective from another treatment center. Insight in OCD is difficult to operationalize and is unlikely to be captured with a single scale. The BABS assesses insight with reference to the individual's predominant symptom, which may lack sensitivity to insight of the condition. Finally, the protocol did not involve a blinded component, as no participant agreed to the potential for their device to be randomly switched off. One participant inadvertently allowed the battery to go flat and experienced an overwhelming exacerbation of OCD symptoms, with a huge surge in anxiety; there was immediate restitution once the battery was charged. Further, here we presented the primary and secondary outcomes of the clinical trial, which do not portray the profound changes the participants experienced in their everyday life. Subjective experiences, psychosocial outcomes, and a lived experience investigation are reported elsewhere.^30^, ^31^

Future trials of DBS therapy for TR‐OCD should implement a multi‐disciplinary adjunct treatment approach targeting several aspects of functional recovery, with consistent programming adjustments. Follow up reports should also consider different aspects of functioning and the patients' perspective to gain a more comprehensive understanding of DBS mechanisms in obsessive compulsive symptomology. Insight should be further investigated within this context, by evaluation of different elements of insight relating to (1) condition, (2) symptom, and (3) treatment, as possible mediators of clinical response. Also, pooling of data in a clinical registry would allow comprehensive analysis to establish if baseline insight can predict individual clinical response. Although a specific clinical predictor of DBS response may not exist for this patient group, pooling multimodal data may identify a set of factors that provide more nuanced prognostic predictions. This approach would allow progression towards personalized precision medicine for this patient group.

Although the improvements described were clinically and personally meaningful, DBS therapy comes with risks from the surgery, device, and/or stimulation. In our cohort, two participants experienced serious transient adverse events from surgical complications, which required medical follow up. Another two participants experienced hypomanic symptoms from increases in stimulation voltage. Hypomania is common in individuals receiving DBS within the dorsal striatum for OCD^69^ and may even signify effectiveness of the therapy.^20^ However, the presence of hypomania complicates increases in stimulation, which are necessary to optimize outcomes. Therefore, a predisposition to hypomania could be screened pre‐operatively and considered in the decision making of the surgical target. Further, tractography‐guided surgical targeting has been implemented in one identified OCD cohort, and led to greater responders, and significantly fewer stimulation related side effects (including hypomania) compared to conventional targeting.^70^ This novel approach shows potential utility in prospective targeting to optimize outcomes and minimize side effects.

## Conclusion

We demonstrated the efficacy of DBS for TR‐OCD and provided further support for the prognostic utility of insight, within a field lacking predictive factors. We encourage a multidisciplinary and biopsychosocial approach in tailoring therapy and evaluating effectiveness in line with personally meaningful goals. DBS, and other neuromodulation therapies should not be seen as a stand‐alone solution, rather a synergistic tool that can provide alleviation from severe distress, allowing individuals to engage in psychotherapy and consolidate DBS mediated changes. The effect size of DBS for TR‐OCD is large, particularly when considering those of conventional therapies and the refractoriness of individuals undergoing DBS.

## Disclosure statement

The authors declare no conflict of interest.

## Author contributions

SR, DC, CG, MC, PM, JO, PB contributed to the conception and design, SR, DC, CG, MC, PM, JO, PB, DM, TP, NA contributed to the acquisition and analysis of data, NA contributed to drafting the manuscript and figures.

## Supporting information


**Supplementary Figure S1.** Montgomery Algorithm.

**Supplementary S2.** Lead localizations in the left hemisphere within 2D axial, coronal and sagittal planes of responders (blue dots) and non‐responders (purple dots).

**Supplementary S3.** Lead localizations in the right hemisphere within 2D axial, coronal and sagittal planes of responders (blue dots) and non‐responders (purple dots).

**Supplementary S4.** Volume of tissue activated estimates according to final stimulation parameters for participants 1–8.

**Supplementary S5.** Video representation of lead localizations of responders (blue) and non‐responders (purple).

## Data Availability

De‐identified data is available upon request.

## References

[1] American Psychiatric Association, D.S.M.T.F., Diagnostic and statistical manual of mental disorders: DSM‐5, ed. A. American Psychiatric and D.S.M.T.F. American Psychiatric Association . Arlington. American Psychiatric Association, VA, 2013.

[2] Hollander E , Doernberg E , Shavitt R *et al*. The cost and impact of compulsivity: A research perspective. Eur Neuropsychopharmacol 2016; 26: 800–809. 27235690 10.1016/j.euroneuro.2016.02.006

[3] Pallanti S , Quercioli L . Treatment‐refractory obsessive‐compulsive disorder: Methodological issues, operational definitions and therapeutic lines. Prog Neuropsychopharmacol Biol Psychiatry 2006; 30: 400–412. 16503369 10.1016/j.pnpbp.2005.11.028

[4] Hirschtritt ME , Bloch MH , Mathews CA . Obsessive‐compulsive disorder: Advances in diagnosis and treatment. Jama 2017; 317: 1358–1367. 28384832 10.1001/jama.2017.2200

[5] Menzies L , Chamberlain SR , Laird AR , Thelen SM , Sahakian BJ , Bullmore ET . Integrating evidence from neuroimaging and neuropsychological studies of obsessive‐compulsive disorder: The orbitofronto‐striatal model revisited. Neurosci Biobehav Rev 2008; 32: 525–549. 18061263 10.1016/j.neubiorev.2007.09.005 PMC2889493

[6] Nakao T , Okada K , Kanba S . Neurobiological model of obsessive‐compulsive disorder: Evidence from recent neuropsychological and neuroimaging findings. Psychiatry Clin Neurosci 2014; 68: 587–605. 24762196 10.1111/pcn.12195

[7] Denys D , Schuurman PR . Basic principles of deep brain stimulation in deep brain stimulation, a new frontier in psychiatry. Springer, Berlin, 2012.

[8] Mar‐Barrutia L , Real E , Segalás C , Bertolín S , Menchón JM , Alonso P . Deep brain stimulation for obsessive‐compulsive disorder: A systematic review of worldwide experience after 20 years. World J Psychiatry 2021; 11: 659. 34631467 10.5498/wjp.v11.i9.659 PMC8474989

[9] Gadot R , Najera R , Hirani S *et al*. Efficacy of deep brain stimulation for treatment‐resistant obsessive‐compulsive disorder: Systematic review and meta‐analysis. J Neurol Neurosurg Psychiatry 2022; 93: 1166–1173. 10.1136/jnnp-2021-328738 36127157

[10] Acevedo N , Rossell S . Deep brain stimulation for treatment‐refractory obsessive‐compulsive disorder should be an accepted therapy in Australia. Aust New Zeal J Psychiatry 2021; 56: 301–302. 10.1177/00048674211049344 34585969

[11] van Wingen G , Bergfeld I , de Koning P *et al*. Comment to: Deep brain stimulation for refractory obsessive‐compulsive disorder (OCD): Emerging or established therapy? Mol Psychiatry 2022; 27: 1276–1277. 34992236 10.1038/s41380-021-01411-8

[12] Visser‐Vandewalle V , Andrade P , Mosley PE *et al*. Deep brain stimulation for obsessive–compulsive disorder: A crisis of access. Nat Med 2022; 28: 1529–1532. 35840727 10.1038/s41591-022-01879-z

[13] Alonso P , Cuadras D , Gabriëls L *et al*. Deep brain stimulation for obsessive‐compulsive disorder: A meta‐analysis of treatment outcome and predictors of response. PLoS One 2015; 10: e0133591. 26208305 10.1371/journal.pone.0133591 PMC4514753

[14] Graat I , Mocking RJT , de Koning P *et al*. Predicting response to vALIC deep brain stimulation for refractory obsessive‐compulsive disorder. J Clin Psychiatry 2021; 82: 37759–37765. 10.4088/JCP.20m13754 34727424

[15] Raymaekers S , Hendrickx S , Raymaekers S , Gabriëls L , Nuttin B . Long‐term electrical stimulation of bed nucleus of stria terminalis for obsessive‐compulsive disorder. Mol Psychiatry 2017; 22: 931–934. 27480493 10.1038/mp.2016.124

[16] Denys D , Mantione M , Figee M *et al*. Deep brain stimulation of the nucleus Accumbens for treatment‐refractory obsessive‐compulsive disorder. Arch Gen Psychiatry 2010; 67: 1061–1068. 20921122 10.1001/archgenpsychiatry.2010.122

[17] Graat I , Mocking R , Figee M *et al*. Long‐term outcome of deep brain stimulation of the ventral part of the anterior limb of the internal capsule in a cohort of 50 patients with treatment‐refractory obsessive‐compulsive disorder. Biol Psychiatry 2020; 90: 714–720. 33131717 10.1016/j.biopsych.2020.08.018

[18] Li N , Baldermann JC , Kibleur A *et al*. A unified connectomic target for deep brain stimulation in obsessive‐compulsive disorder. Nat Commun 2020; 11: 3364. 32620886 10.1038/s41467-020-16734-3 PMC7335093

[19] Acevedo N , Bosanac P , Pikoos T , Rossell S , Castle D . Therapeutic neurostimulation in obsessive‐compulsive and related disorders: A systematic review. Brain Sci 2021; 11: 948. 34356182 10.3390/brainsci11070948 PMC8307974

[20] Denys D , Graat I , Mocking R *et al*. Efficacy of deep brain stimulation of the ventral anterior limb of the internal capsule for refractory obsessive‐compulsive disorder: A clinical cohort of 70 patients. Am J Psychiatry 2020; 177: 265–271. 31906709 10.1176/appi.ajp.2019.19060656

[21] Mantione M , Nieman DH , Figee M , Denys D . Cognitive‐behavioural therapy augments the effects of deep brain stimulation in obsessive‐compulsive disorder. Psychol Med 2014; 44: 3515–3522. 25065708 10.1017/S0033291714000956

[22] Mosley PE , Windels F , Morris J *et al*. A randomised, double‐blind, sham‐controlled trial of deep brain stimulation of the bed nucleus of the stria terminalis for treatment‐resistant obsessive‐compulsive disorder. Transl Psychiatry 2021; 11: 190–207. 33782383 10.1038/s41398-021-01307-9 PMC8007749

[23] Winter L , Saryyeva A , Schwabe K *et al*. Long‐term deep brain stimulation in treatment‐resistant obsessive‐compulsive disorder: Outcome and quality of life at four to eight years follow‐up. Neuromodulation 2020; 24: 324–330. 32667114 10.1111/ner.13232

[24] Montgomery EB . Programming, Principles and Practice. Deep Brain Stimulation Programming. Oxford University Press, New York, 2010.

[25] Acevedo N , Castle D , Groves C , Bosanac P , Mosley PE , Rossell S . Clinical recommendations for the care of people with treatment‐refractory obsessive‐compulsive disorder when undergoing deep brain stimulation. Aust New Zeal J Psychiatry 2022; 56: 1219–1225. 10.1177/00048674221100947 35603702

[26] Goodman WK , Price LH , Rasmussen SA *et al*. The Yale‐Brown obsessive compulsive scale: I. Development, use, and reliability. Arch Gen Psychiatry 1989; 46: 1006–1011. 2684084 10.1001/archpsyc.1989.01810110048007

[27] Hamilton M . A rating scale for depression. J Neurol Neurosurg Psychiatry 1960; 23: 56–62. 14399272 10.1136/jnnp.23.1.56 PMC495331

[28] Hamilton M . The assessment of anxiety states by rating. Br J Med Psychol 1959; 32: 50–55. 13638508 10.1111/j.2044-8341.1959.tb00467.x

[29] Eisen J , Phillips KA , Baer L *et al*. The Brown assessment of beliefs scale: Reliability and validity. Am J Psychiatry 1998; 155: 102–108. 9433346 10.1176/ajp.155.1.102

[30] Acevedo N , Castle DJ , Bosanac P , Groves C , Rossell SL . Patient feedback and psychosocial outcomes of deep brain stimulation in people with obsessive–compulsive disorder. J Clin Neurosci 2023; 112: 80–85. 37119742 10.1016/j.jocn.2023.04.012

[31] Acevedo N , Castle D , Bosanac P , Rossell S . Phenomenological changes associated with deep brain stimulation for obsessive compulsive disorder: A cognitive appraisal model of recovery. Brain Sci 2023; 13: 1444. 37891812 10.3390/brainsci13101444 PMC10605199

[32] Horn A , Li N , Dembek TA *et al*. Lead‐DBS v2: Towards a comprehensive pipeline for deep brain stimulation imaging. Neuroimage 2019; 184: 293–316. 30179717 10.1016/j.neuroimage.2018.08.068 PMC6286150

[33] Avants BB , Tustison N , Song G . Advanced normalization tools (ANTS). Insight j 2009; 2: 1–35.

[34] Husch A , Petersen MV , Gemmar P , Goncalves J , Hertel F . PaCER‐A fully automated method for electrode trajectory and contact reconstruction in deep brain stimulation. NeuroImage: Clin 2018; 17: 80–89. 29062684 10.1016/j.nicl.2017.10.004 PMC5645007

[35] Pauli WM , Nili AN , Tyszka JM . A high‐resolution probabilistic in vivo atlas of human subcortical brain nuclei. Sci Data 2018; 5: 1–13. 29664465 10.1038/sdata.2018.63 PMC5903366

[36] van Westen M , Rietveld E , Bergfeld IO *et al*. Optimizing Deep Brain Stimulation Parameters in Obsessive–Compulsive Disorder. Neuromodulation: Technology at the Neural Interface, 2020. 10.1111/ner.13243 PMC7984355 33128489

[37] Markova IS , Jaafari N , Berrios GE . Insight and obsessive‐compulsive disorder: A conceptual analysis. Psychopathology 2009; 42: 277–282. 19609097 10.1159/000228836

[38] Alonso P , Menchón JM , Segalàs C *et al*. Clinical implications of insight assessment in obsessive‐compulsive disorder. Compr Psychiatry 2008; 49: 305–312. 18396191 10.1016/j.comppsych.2007.09.005

[39] Fontenelle J , da S Santana L , da R Lessa L , da Victoria MS , Mendlowicz MV , Fontenelle LF . The concept of insight in patients with obsessive‐compulsive disorder. Rev Brasil Psiquiatria 2010; 32: 77–82. 10.1590/s1516-44462010000100015 20339738

[40] Kishore VR , Samar R , Reddy YCJ , Chandrasekhar CR , Thennarasu K . Clinical characteristics and treatment response in poor and good insight obsessive–compulsive disorder. Eur Psychiatry 2004; 19: 202–208. 15196601 10.1016/j.eurpsy.2003.12.005

[41] Landi P *et al*. Insight in psychiatry and neurology: State of the art, and hypotheses. Harv Rev Psychiatry 2016; 24: 214–228. 27075815 10.1097/HRP.0000000000000083

[42] Matsunaga H , Kiriike N , Matsui T *et al*. Obsessive‐compulsive disorder with poor insight. Comprehens Psychiatry 2002; 43: 150–157. 10.1053/comp.2002.30798 11893994

[43] Türksoy N , Tükel R , Ozdemir O , Karali A . Comparison of clinical characteristics in good and poor insight obsessive–compulsive disorder. J Anxiety Disord 2002; 16: 413–423. 12213036 10.1016/s0887-6185(02)00135-4

[44] Kahn L , Sutton B , Winston HR , Abosch A , Thompson JA , Davis RA . Deep brain stimulation for obsessive‐compulsive disorder: Real world experience post‐FDA‐humanitarian use device approval. Front Psychiatry 2021; 12: 568932. 33868034 10.3389/fpsyt.2021.568932 PMC8044872

[45] Barcia JA *et al*. Personalized striatal targets for deep brain stimulation in obsessive‐compulsive disorder. Brain Stimul 2019; 12: 724–734. 30670359 10.1016/j.brs.2018.12.226

[46] Burdick A , Avecillas‐Chasín JM , Nombela C *et al*. Lack of benefit of accumbens/capsular deep brain stimulation in a patient with both tics and obsessive‐compulsive disorder. Neurocase 2010; 16: 321–330. 20178034 10.1080/13554790903560422

[47] Farrand S , Evans AH , Mangelsdorf S *et al*. Deep brain stimulation for severe treatment‐resistant obsessive‐compulsive disorder: An open‐label case series. Aust New Zeal J Psychiatry 2018; 52: 699–708. 10.1177/0004867417731819 28965430

[48] Franzini A , Messina G , Gambini O *et al*. Deep‐brain stimulation of the nucleus accumbens in obsessive compulsive disorder: Clinical, surgical and electrophysiological considerations in two consecutive patients. Neurol Sci 2010; 31: 353–359. 20127500 10.1007/s10072-009-0214-8

[49] Grant JE , Odlaug BL , Chamberlain SR . Neurocognitive response to deep brain stimulation for obsessive‐compulsive disorder: A case report. Am J Psychiatry 2011; 168: 1338–1339. 10.1176/appi.ajp.2011.11071108 22193674

[50] Huff W , Lenartz D , Schormann M *et al*. Unilateral deep brain stimulation of the nucleus accumbens in patients with treatment‐resistant obsessive‐compulsive disorder: Outcomes after one year. Clin Neurol Neurosurg 2010; 112: 137–143. 20006424 10.1016/j.clineuro.2009.11.006

[51] Huys D , Kohl S , Baldermann JC *et al*. Open‐label trial of anterior limb of internal capsule‐nucleus accumbens deep brain stimulation for obsessive‐compulsive disorder: Insights gained. J Neurol Neurosurg Psychiatry 2019; 90: 805–812. 30770458 10.1136/jnnp-2018-318996

[52] Islam L , Franzini A , Messina G , Scarone S , Gambini O . Deep brain stimulation of the nucleus accumbens and bed nucleus of stria terminalis for obsessive‐compulsive disorder: A case series. World Neurosurg 2015; 83: 657–663. 25527882 10.1016/j.wneu.2014.12.024

[53] Welter ML , Santos JFAD , Clair AH *et al*. Deep brain stimulation of the subthalamic, Accumbens, or caudate nuclei for patients with severe obsessive‐compulsive disorder: A randomized crossover controlled study. Biol Psychiatry 2020; 90: 45–47. 10.1016/j.biopsych.2020.07.013 33012521

[54] Gormezoglu M , van der Vlis TB , Schruers K , Ackermans L , Polosan M , Leentjens AFG . Effectiveness, timing and procedural aspects of cognitive behavioral therapy after deep brain stimulation for therapy‐resistant obsessive compulsive disorder: A systematic review. J Clin Med 2020; 9: 2383–3293. 32722565 10.3390/jcm9082383 PMC7464329

[55] Luyten L , Hendrickx S , Raymaekers S , Gabriëls L , Nuttin B . Electrical stimulation in the bed nucleus of the stria terminalis alleviates severe obsessive‐compulsive disorder. Mol Psychiatry 2016; 21: 1272–1280. 26303665 10.1038/mp.2015.124

[56] Greenberg BD , Gabriels LA , Malone DA Jr *et al*. Deep brain stimulation of the ventral internal capsule/ventral striatum for obsessive‐compulsive disorder: Worldwide experience. Mol Psychiatry 2010; 15: 64–79. 18490925 10.1038/mp.2008.55 PMC3790898

[57] Liebrand LC , Caan MWA , Schuurman PR *et al*. Individual white matter bundle trajectories are associated with deep brain stimulation response in obsessive‐compulsive disorder. Brain Stimul 2019; 12: 353–360. 30522916 10.1016/j.brs.2018.11.014

[58] Guzick A , Hunt PJ , Bijanki KR *et al*. Improving long term patient outcomes from deep brain stimulation for treatment‐refractory obsessive‐compulsive disorder. Expert Rev Neurother 2020; 20: 95–107. 31730752 10.1080/14737175.2020.1694409 PMC7227118

[59] Díaz CVT , Treu S , Strange B *et al*. Deep brain stimulation of the nucleus Accumbens, ventral striatum, or internal capsule targets for medication‐resistant obsessive‐compulsive disorder: A multicenter study. World Neurosurg 2021; 155: e168–e176. 34403796 10.1016/j.wneu.2021.08.039

[60] Smith AH , Allison KSC , Waters C *et al*. Replicable effects of deep brain stimulation for obsessive‐compulsive disorder. Brain Stimul 2021; 14: 1–3. 33130018 10.1016/j.brs.2020.10.016

[61] Lee PS , Weiner GM , Corson D *et al*. Outcomes of interventional‐MRI versus microelectrode recording‐guided subthalamic deep brain stimulation. Front Neurol 2018; 9: 241. 29695996 10.3389/fneur.2018.00241 PMC5904198

[62] Baldermann JC , Melzer C , Zapf A *et al*. Connectivity profile predictive of effective deep brain stimulation in obsessive‐compulsive disorder. Biol Psychiatry 2019; 85: 735–743. 30777287 10.1016/j.biopsych.2018.12.019

[63] Gupta A , Khanna S , Jain R . Deep brain stimulation of ventral internal capsule for refractory obsessive‐compulsive disorder. Indian J Psychiatry 2019; 61: 532–536. 31579146 10.4103/psychiatry.IndianJPsychiatry_222_16 PMC6767810

[64] Nuttin BJ , Gabriëls LA , Cosyns PR *et al*. Long‐term electrical capsular stimulation in patients with obsessive‐compulsive disorder. Neurosurgery 2003; 52: 1263–1272 discussion 1272–4. 12762871 10.1227/01.neu.0000064565.49299.9a

[65] Suetens K , Nuttin B , Gabriëls L , Van Laere K *et al*. Differences in metabolic network modulation between capsulotomy and deep‐brain stimulation for refractory obsessive‐compulsive disorder. J Nucl Med 2014; 55: 951–959. 24722531 10.2967/jnumed.113.126409

[66] Tyagi H , Apergis‐Schoute AM , Akram H *et al*. A randomized trial directly comparing ventral capsule and anteromedial subthalamic nucleus stimulation in obsessive‐compulsive disorder: Clinical and imaging evidence for dissociable effects. Biol Psychiatry 2019; 85: 726–734. 30853111 10.1016/j.biopsych.2019.01.017 PMC6467837

[67] Müller S , van Oosterhout A , Bervoets C *et al*. Concerns about psychiatric neurosurgery and how they can Be overcome: Recommendations for responsible research. Neuroethics 2022; 15: 1–26.

[68] Mosley PE , Velakoulis D , Farrand S *et al*. Deep brain stimulation for treatment‐refractory obsessive‐compulsive disorder should be an accepted therapy in Australia. Aust N Z J Psychiatry 2021; 56: 430–436. 34263654 10.1177/00048674211031482

[69] Martinho FP , Duarte GS , Couto FSD . Efficacy, effect on mood symptoms, and safety of deep brain stimulation in refractory obsessive‐compulsive disorder: A systematic review and meta‐analysis. J Clin Psychiatry 2020; 81: 4067–4077. 10.4088/JCP.19r12821 32459406

[70] Graat I , Mocking RJT , Liebrand LC *et al*. Tractography‐based versus anatomical landmark‐based targeting in vALIC deep brain stimulation for refractory obsessive‐compulsive disorder. Mol Psychiatry 2022; 27: 5206–5216. 36071109 10.1038/s41380-022-01760-y

