# Supplementary material for: Clinical outcomes of deep brain stimulation for obsessive‐compulsive disorder: Insight as a predictor of symptom changes
Source: Psychiatry Clin Neurosci. 2023 Dec 5;78(2):131–41. doi: 10.1111/pcn.13619 (PMC10952286; doi:10.1111/pcn.13619)
Supplement: Supplementary file 1 — Supplementary Figure S1. Montgomery Algorithm. [file PCN-78-131-s004.docx]

DBS programming relied on the Montgomery algorithm for adjusting stimulation parameters (Montgomery, 2010), see Supplementary Figure 1. The algorithm uses a systematic approach that commences with monopolar stimulation (DBS lead contacts as cathodes and the implantable pulse generator (IPG) as anode), then wide bipolar stimulation (both anode and cathode contacts on the lead, with 2 contacts between), then multipolar stimulation (multiple cathode contacts on the lead, and anode as another contact on the lead or the IPG). Each stimulation parameter starts at the lowest in the range expected for therapeutic benefit, i.e. frequency of 130Hz, pulse width of 90µs, and intensity commences at 1V, with increments of 0.5V. The voltage (or current) is the first parameter to be increased, whilst avoiding exceeding the battery voltage, or if necessary, exceeding twice the battery voltage. Voltage is increased until therapeutic benefit or a limiting side effect is reached. If the latter occurs, the algorithm is used to select a new electrode configuration, and the process to select stimulation parameters is reinitialized. If therapeutic benefit is not reached, the frequency is increased. If no therapeutic benefit is reached, the voltage and frequency are decreased and the pulse width is increased.


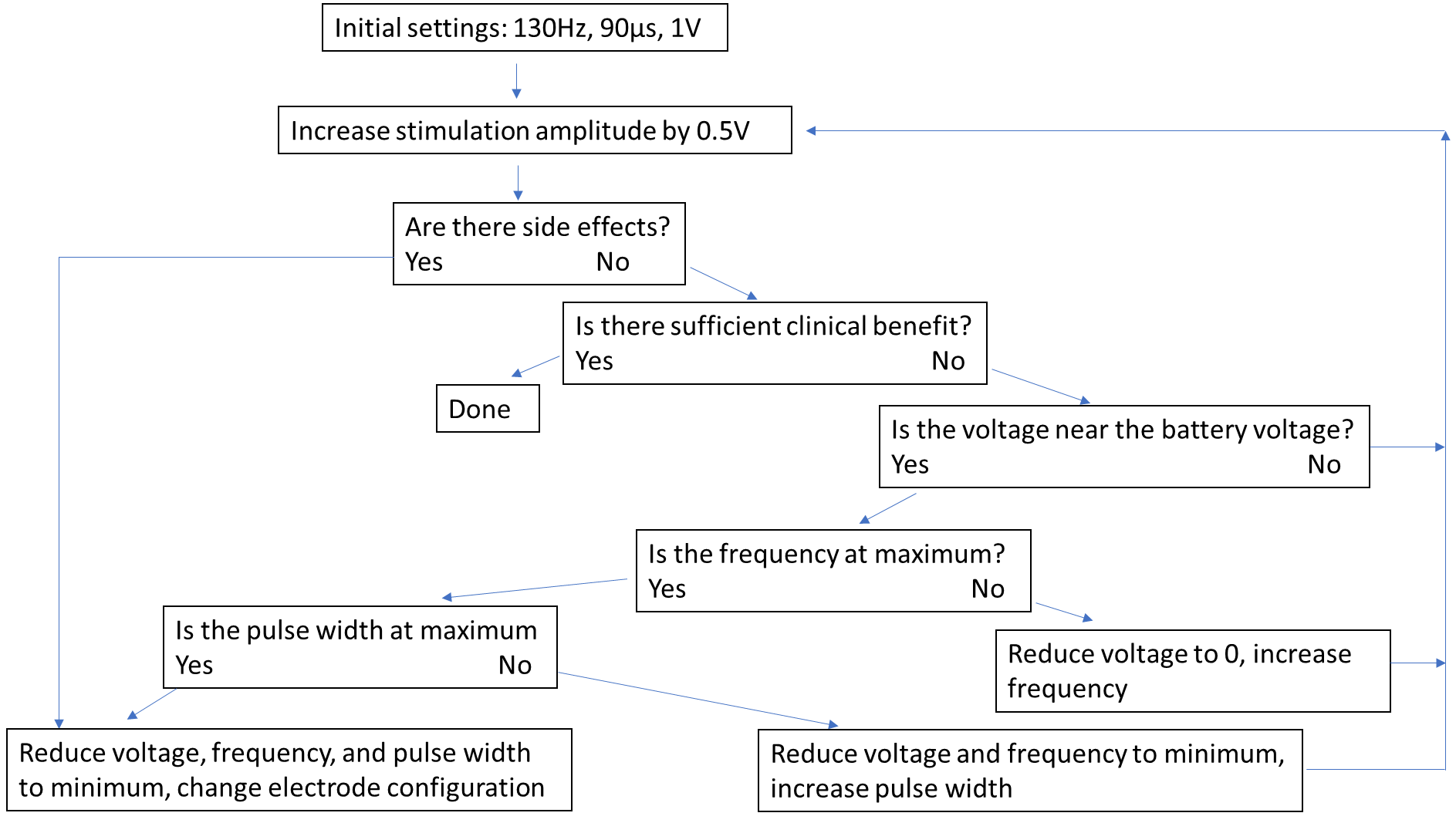


Reference: *MONTGOMERY, E. B. 2010. Montgomery (2010) DBS programming, principles and practice, New York, Oxford University Press.*
