# Supplementary figures and images for: Clinical outcomes of deep brain stimulation for obsessive‐compulsive disorder: Insight as a predictor of symptom changes
Source: Psychiatry Clin Neurosci. 2023 Dec 5;78(2):131–41. doi: 10.1111/pcn.13619 (PMC10952286; doi:10.1111/pcn.13619)

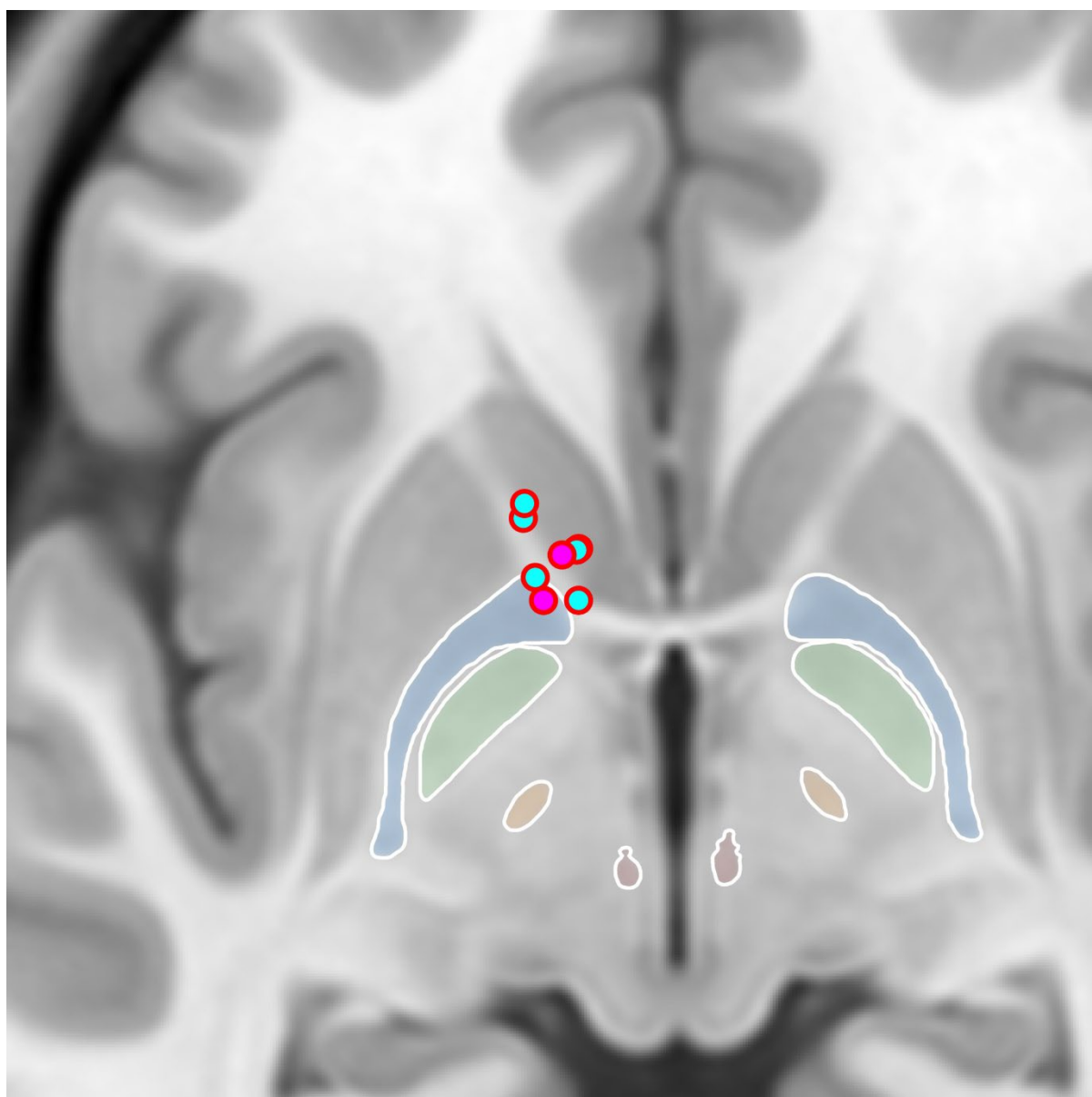

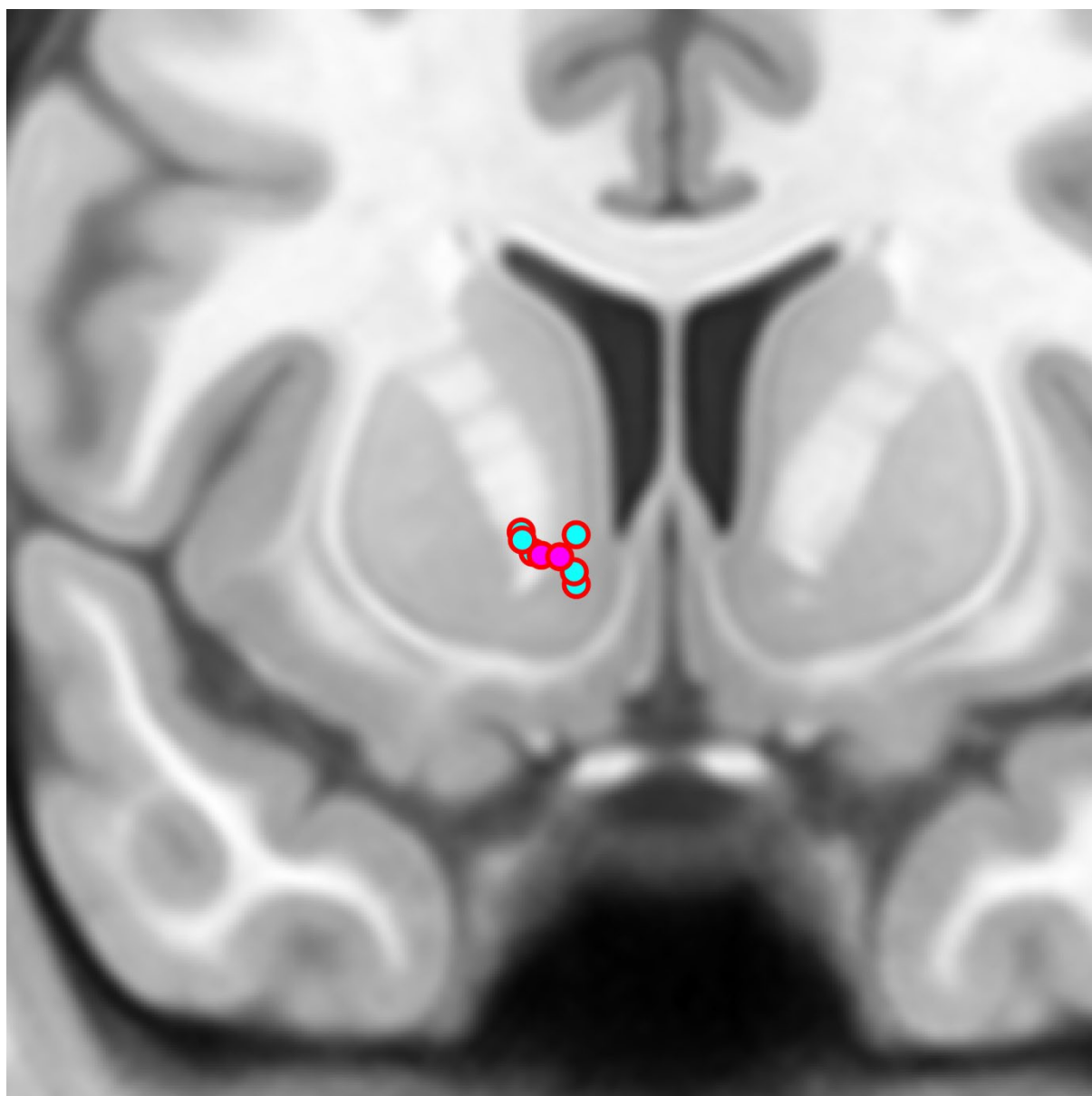

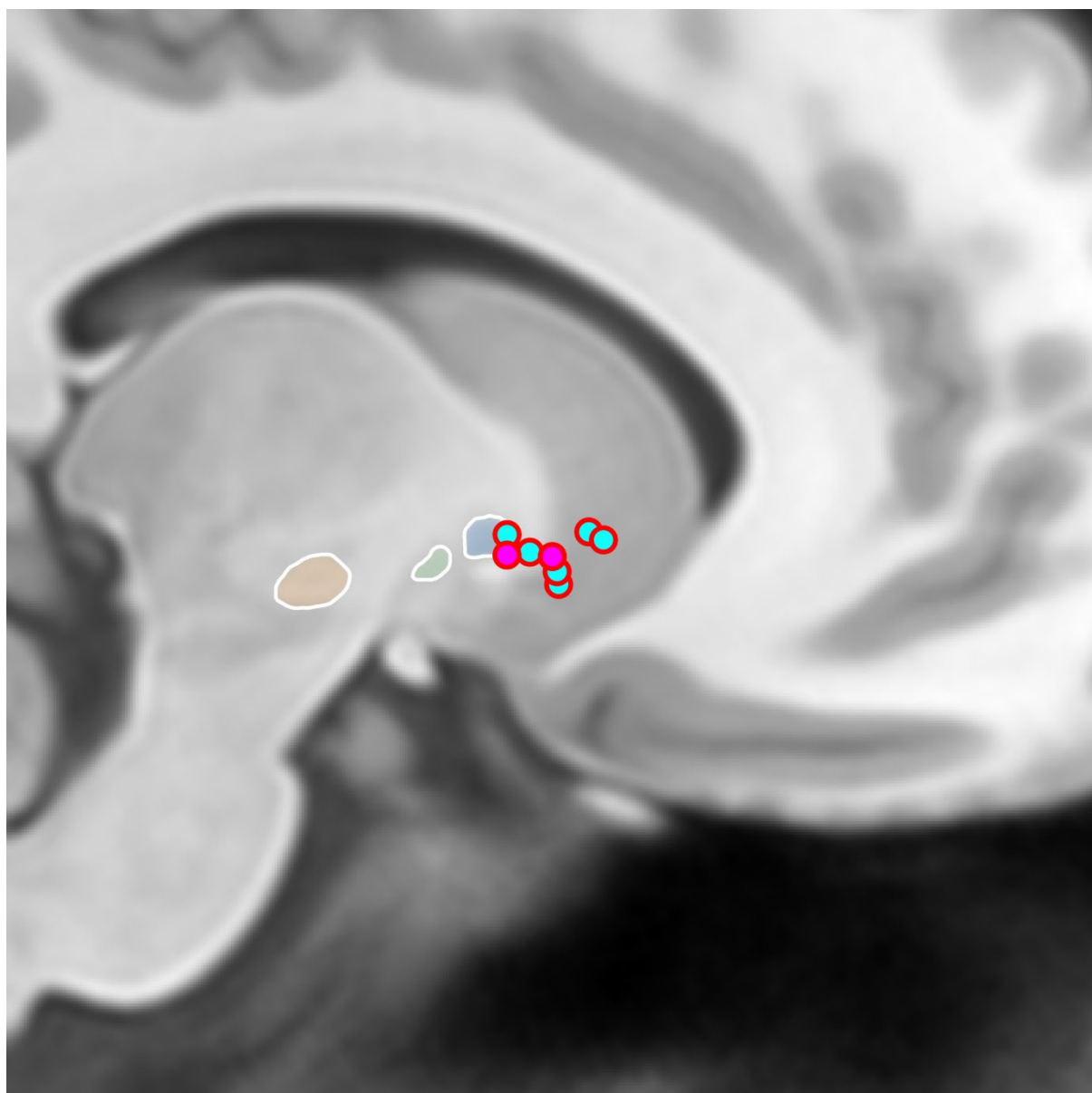

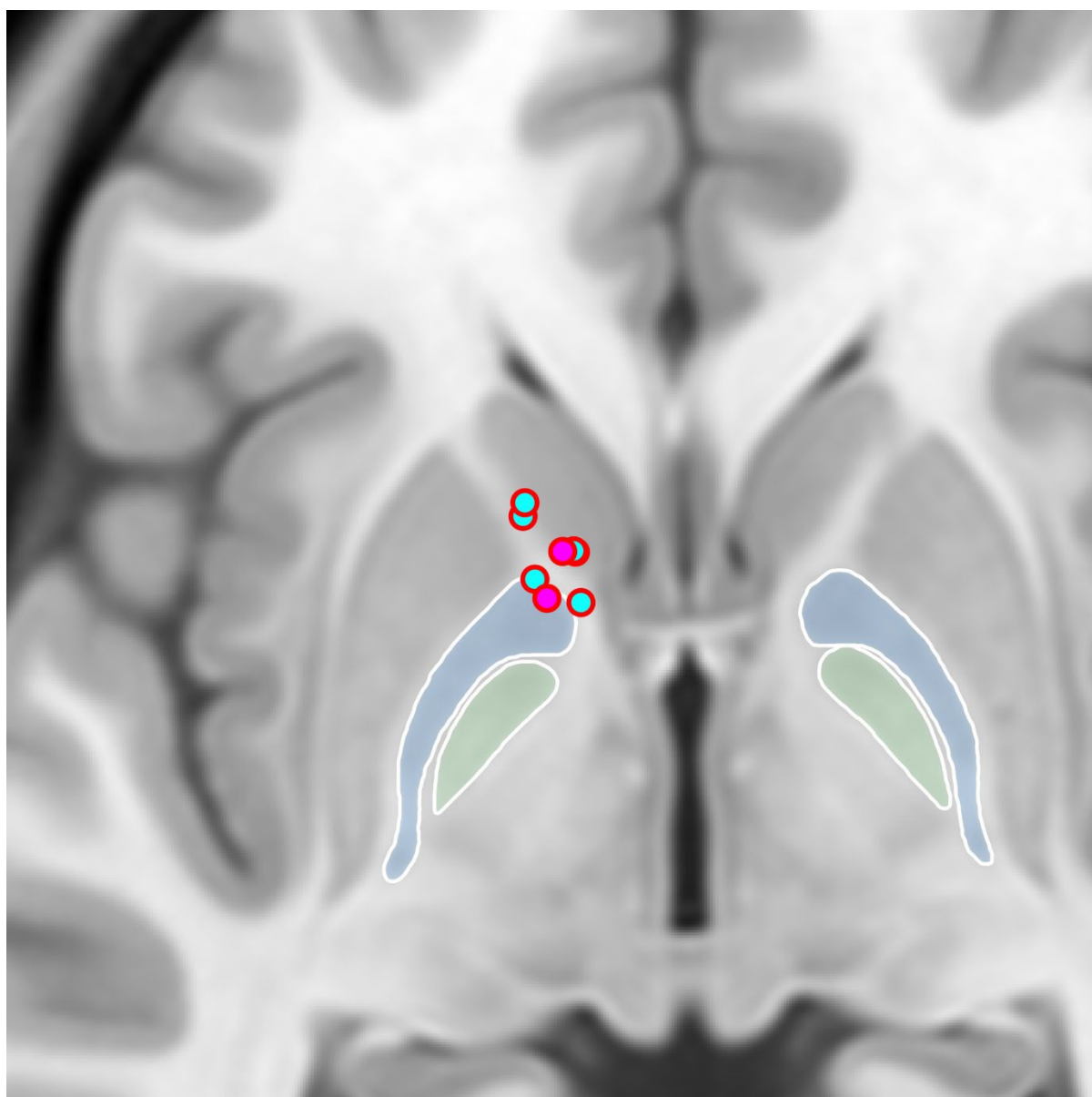

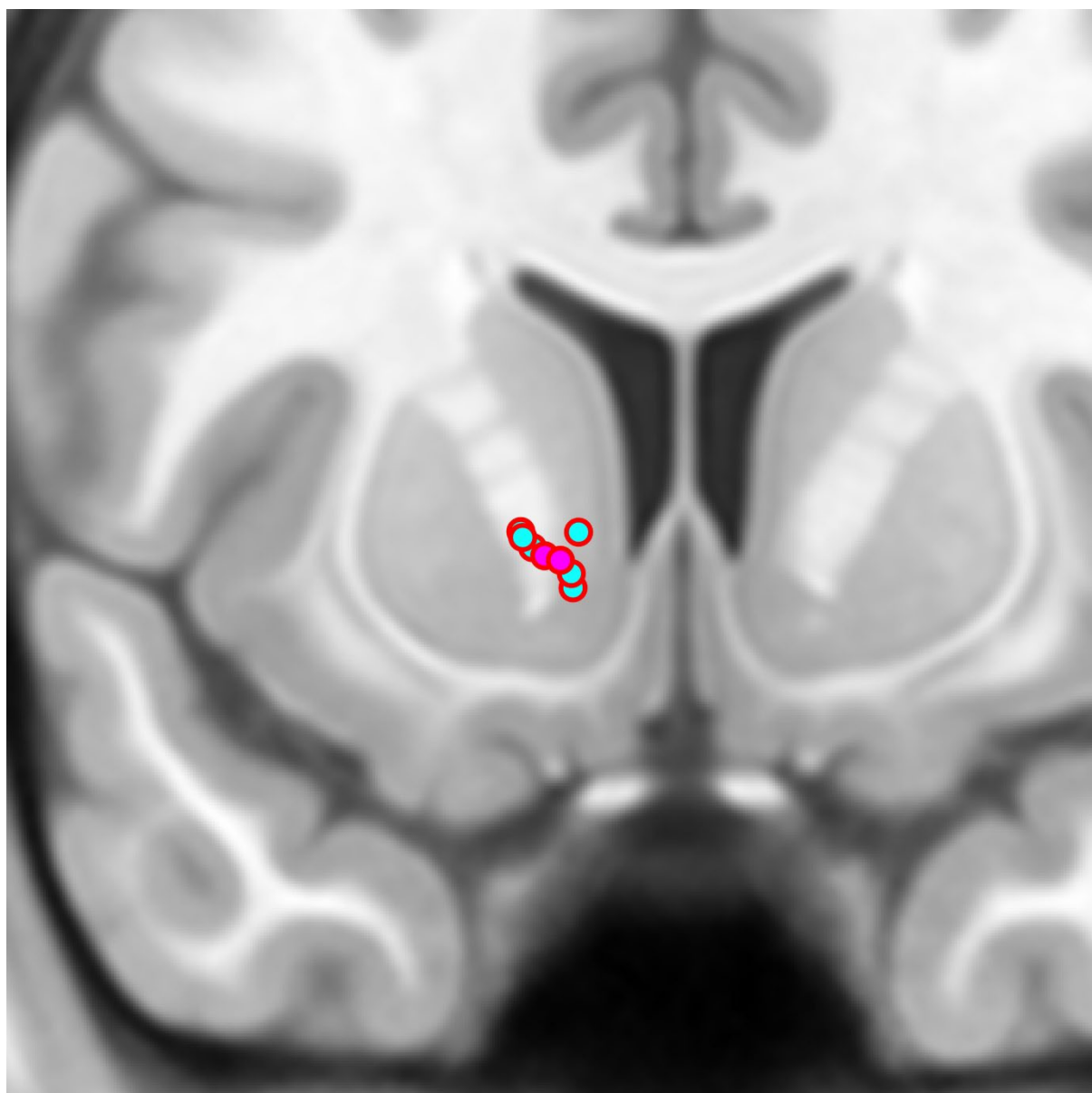

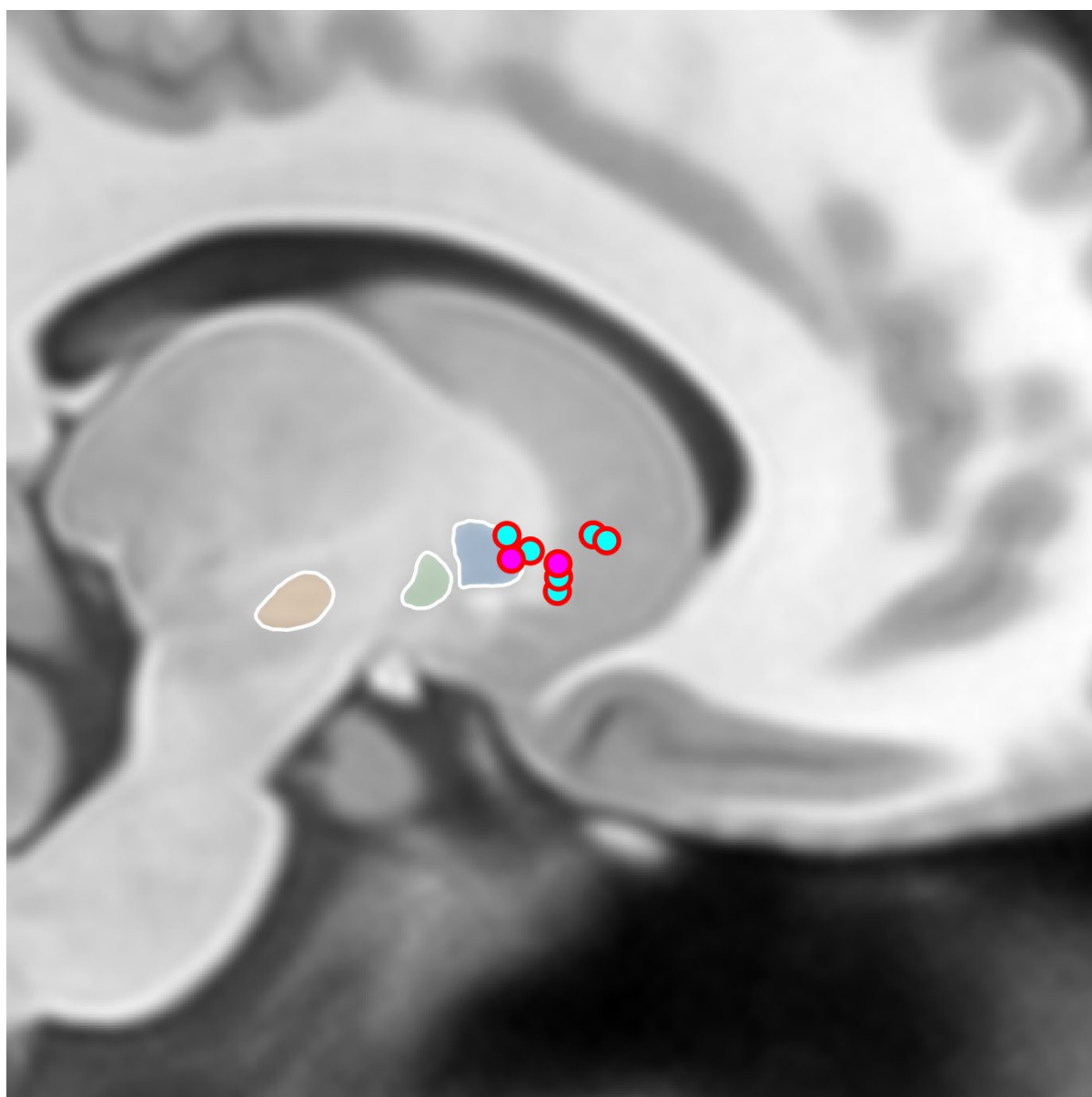

Supplement: Supplementary file 2 — Supplementary S2. Lead localizations in the left hemisphere within 2D axial, coronal and sagittal planes of responders (blue dots) and non‐responders (purple dots). [file PCN-78-131-s003.pdf]

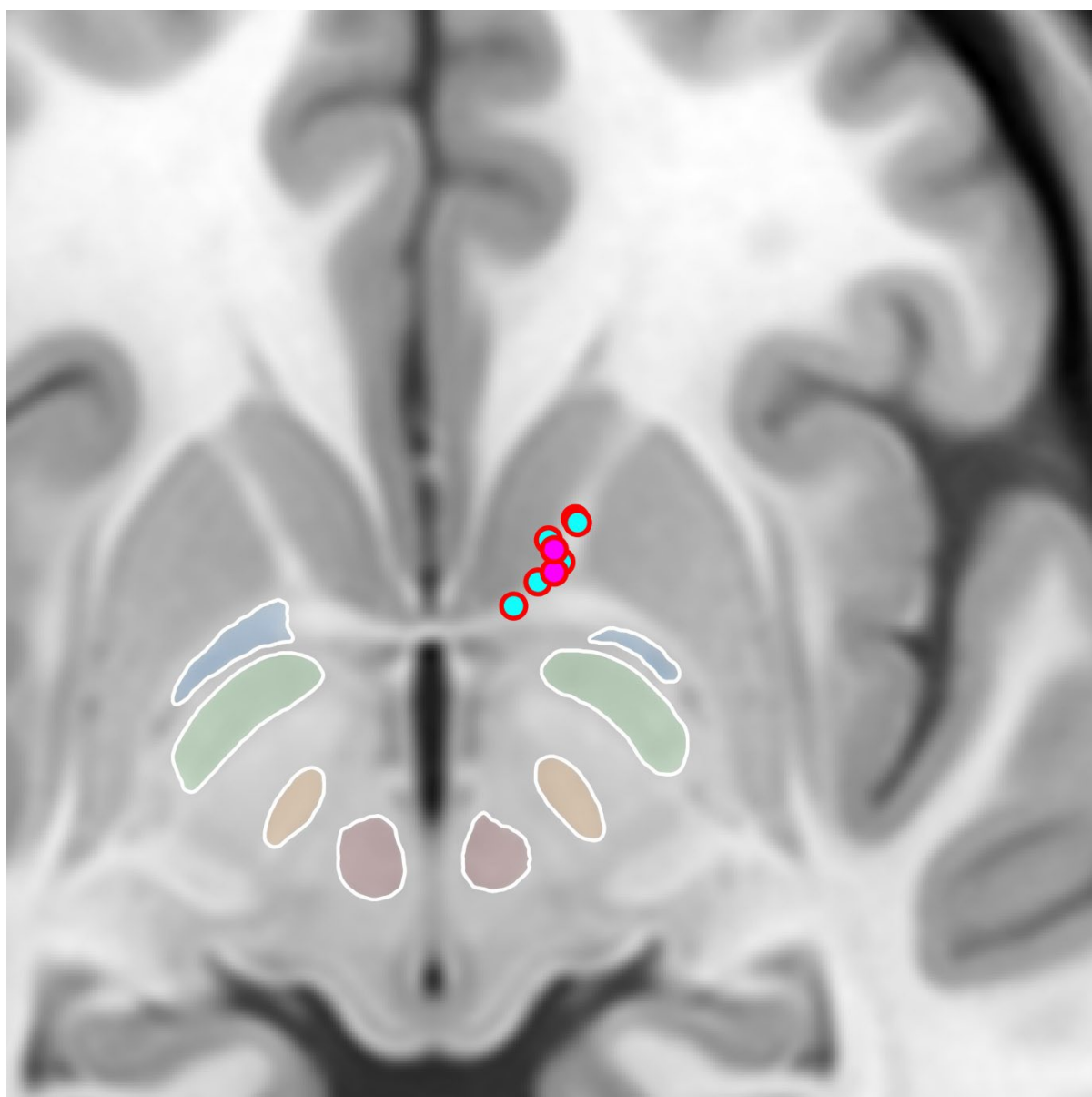

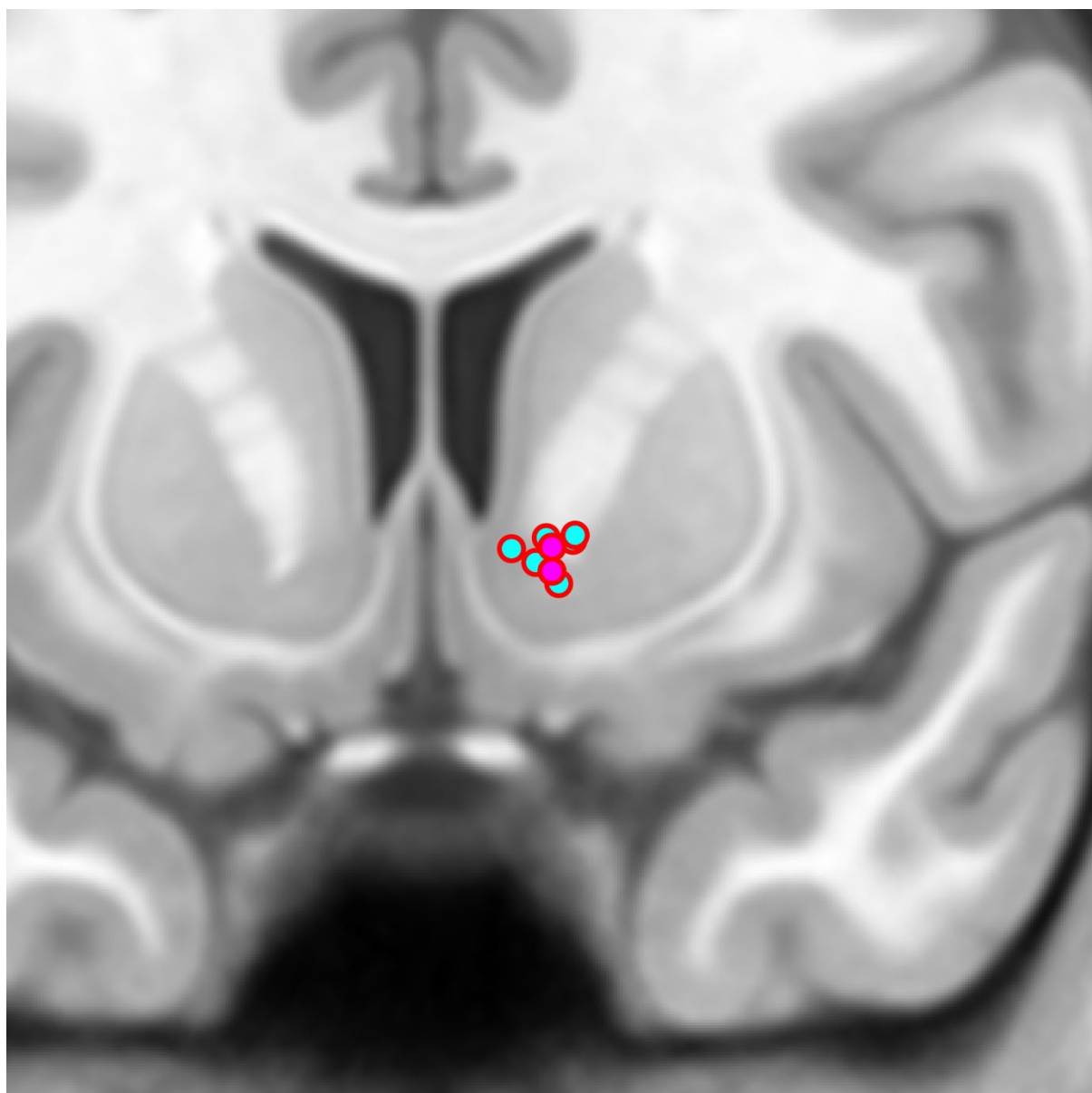

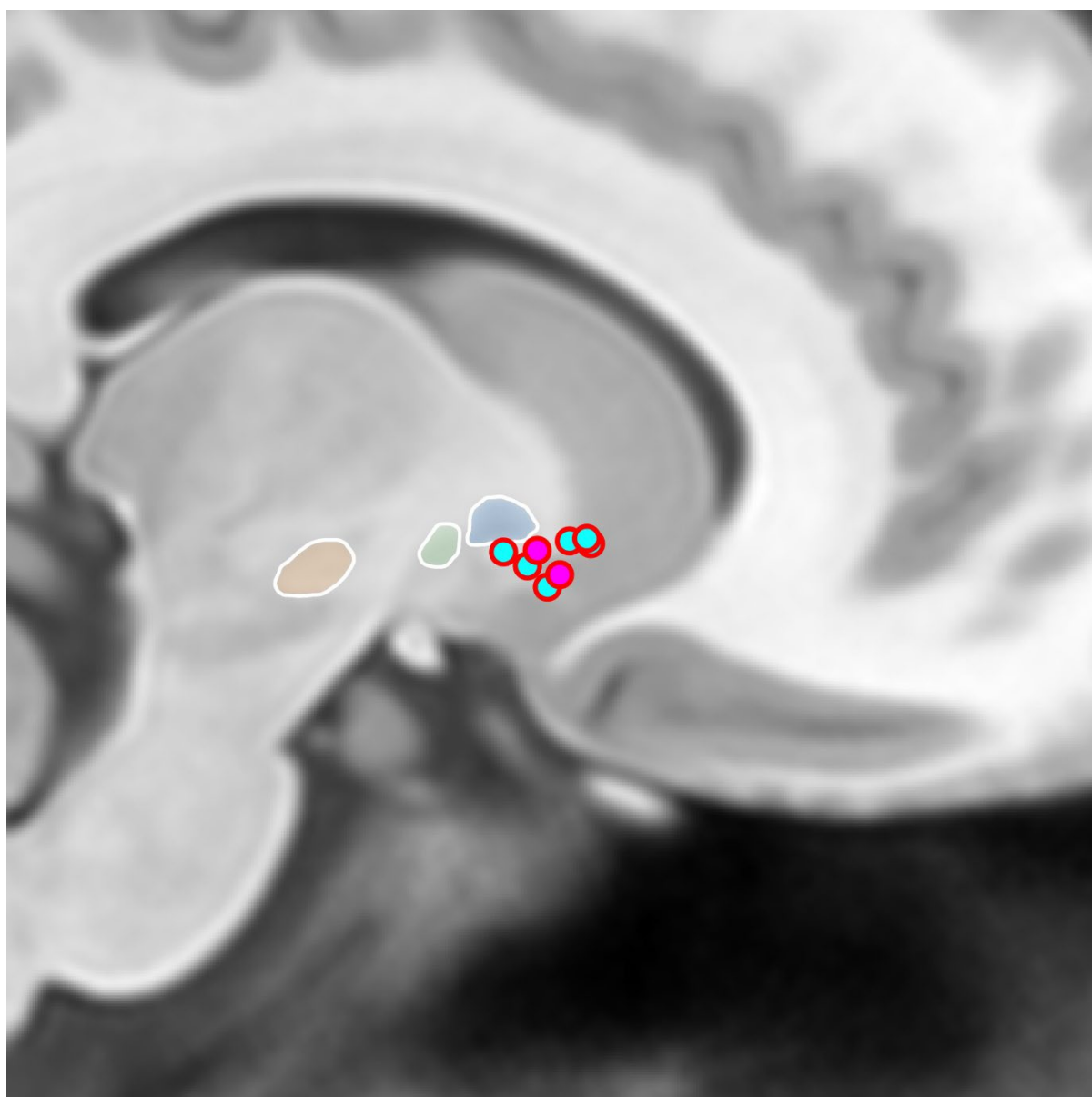

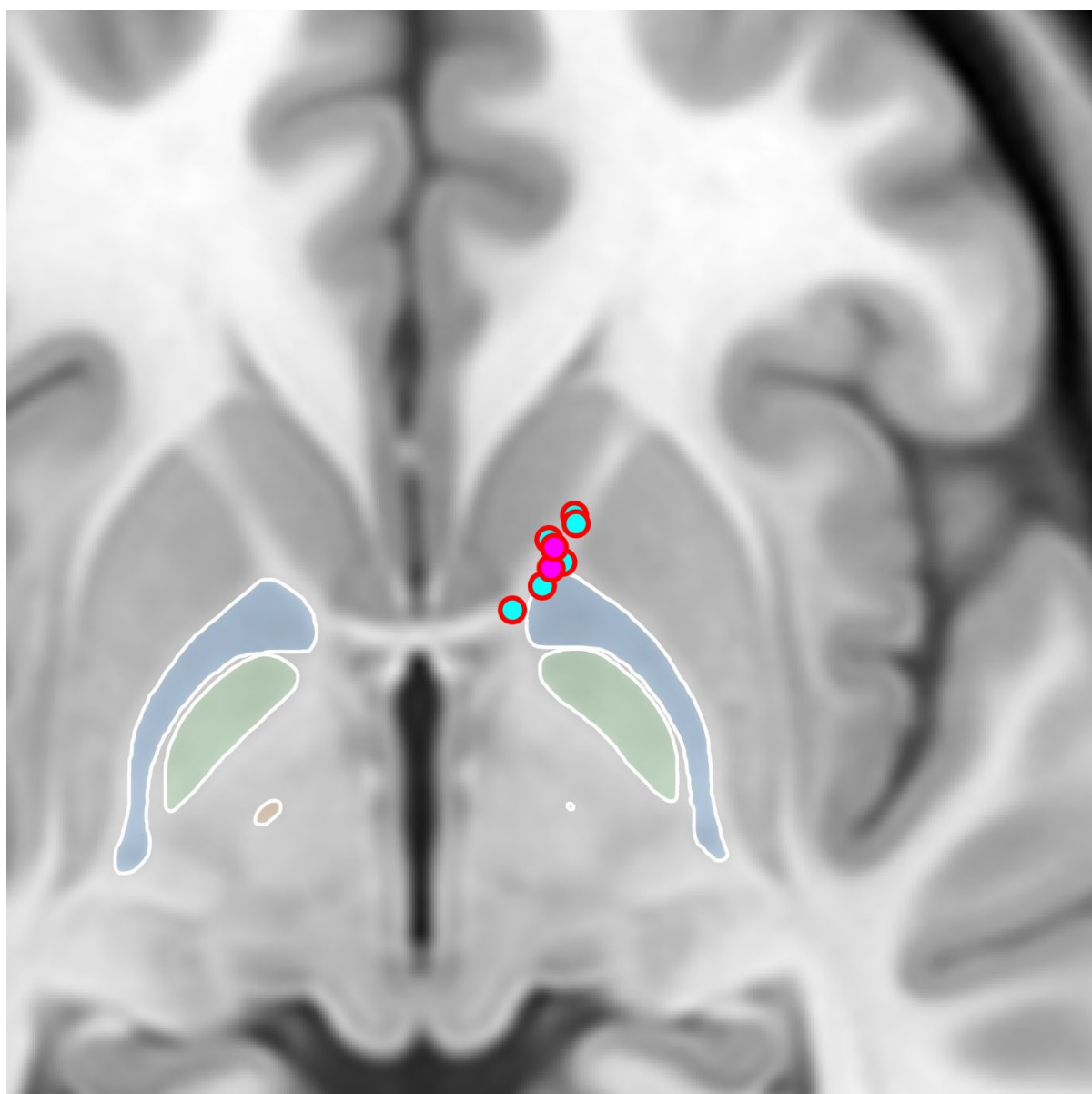

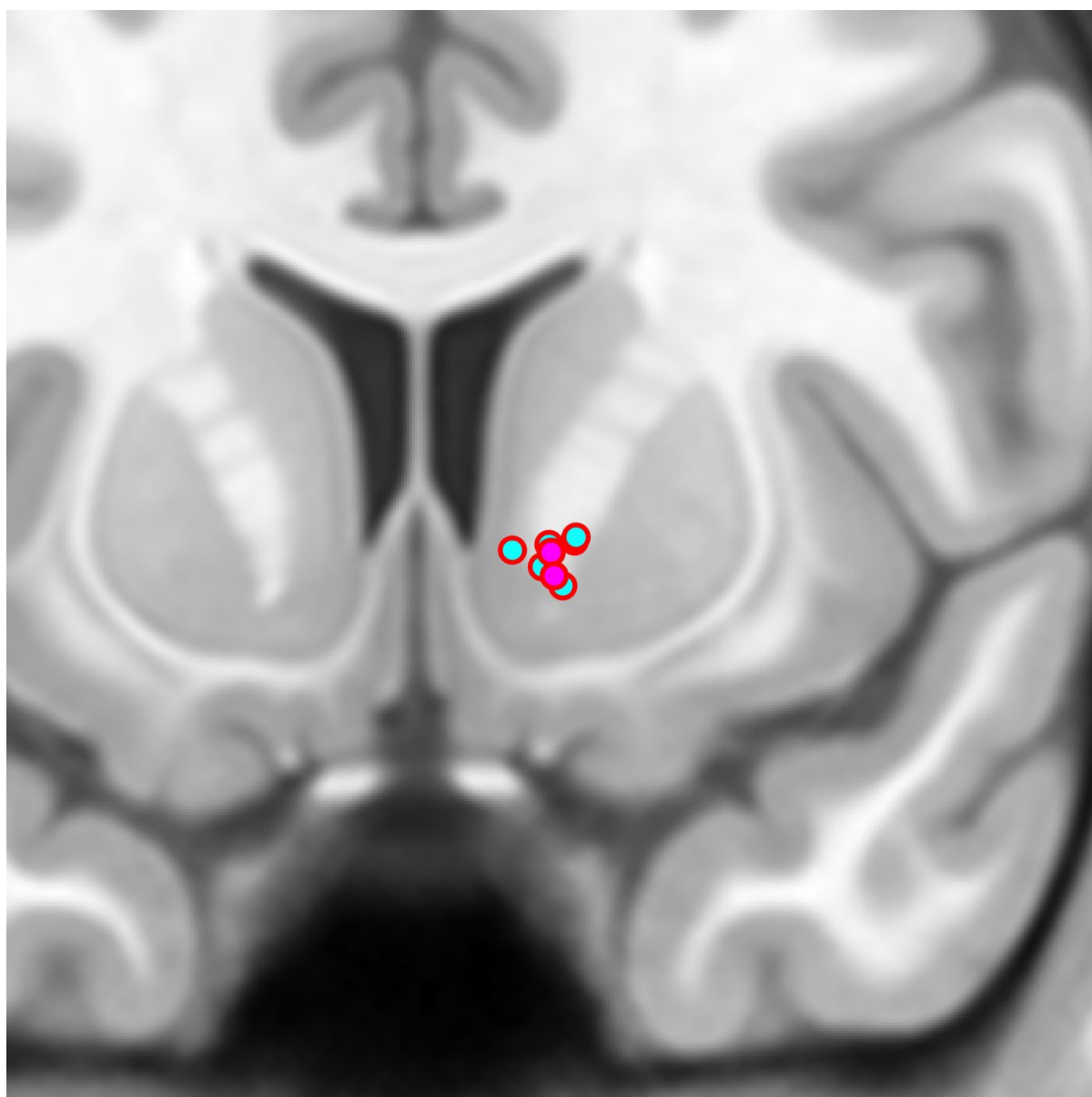

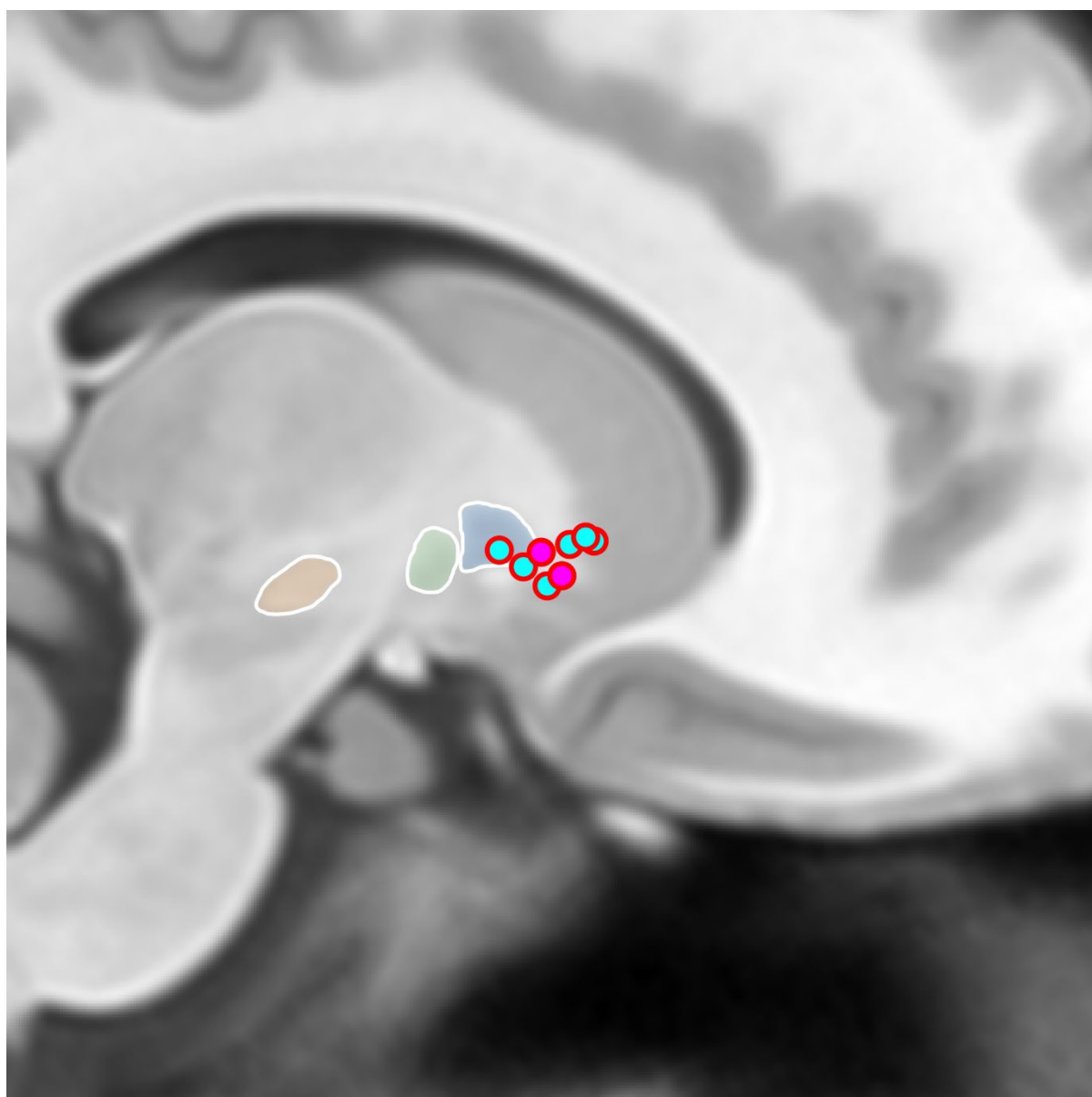

Supplement: Supplementary file 3 — Supplementary S3. Lead localizations in the right hemisphere within 2D axial, coronal and sagittal planes of responders (blue dots) and non‐responders (purple dots). [file PCN-78-131-s005.pdf]

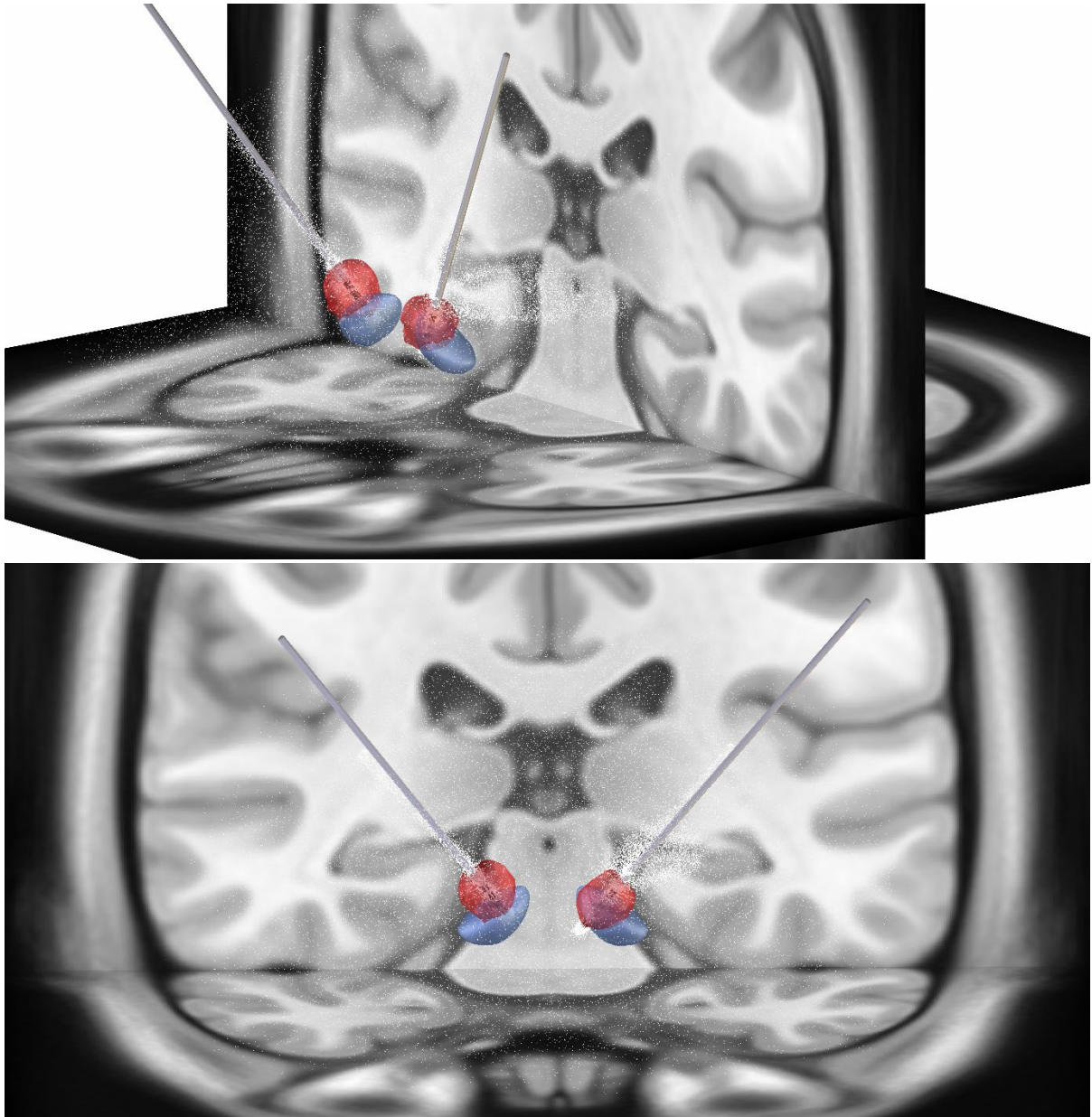

P1

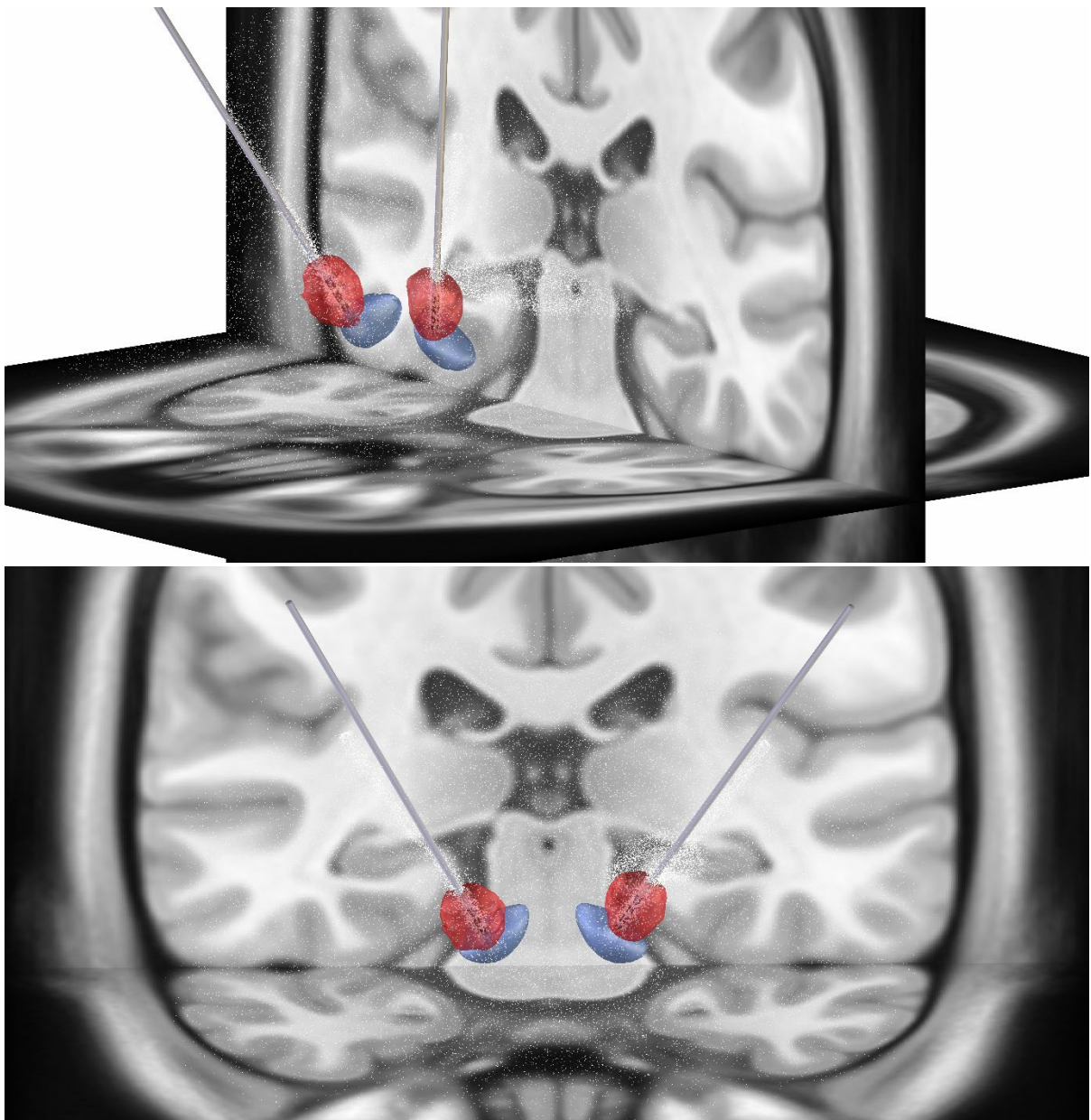

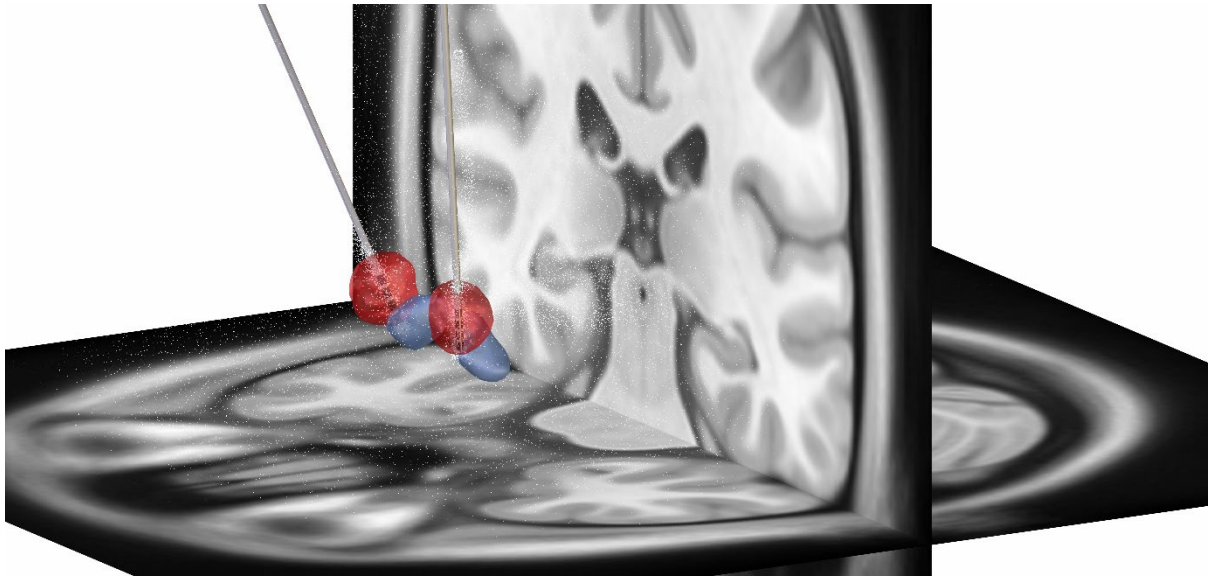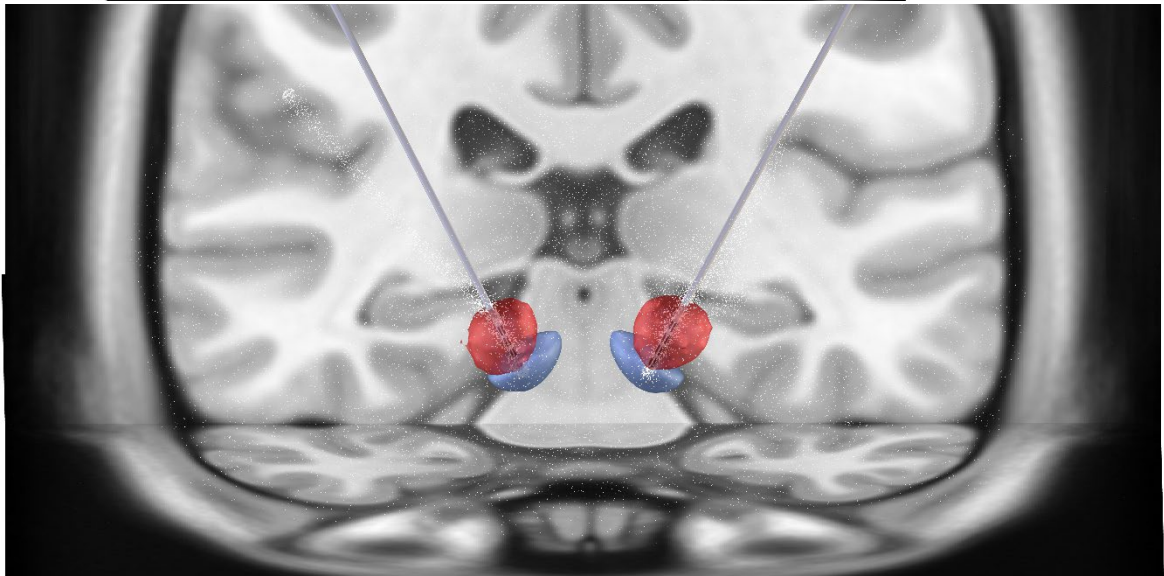

P3

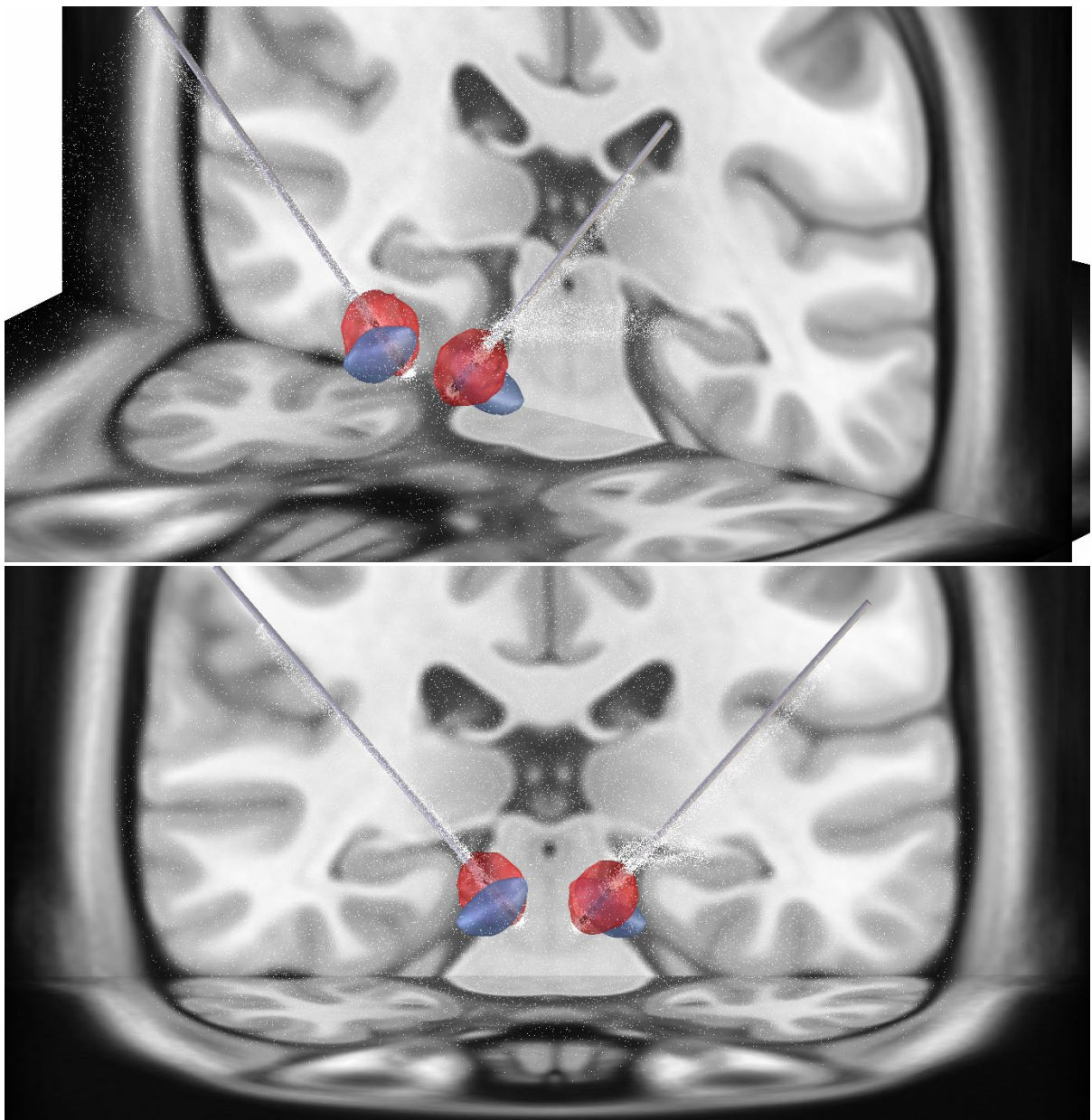

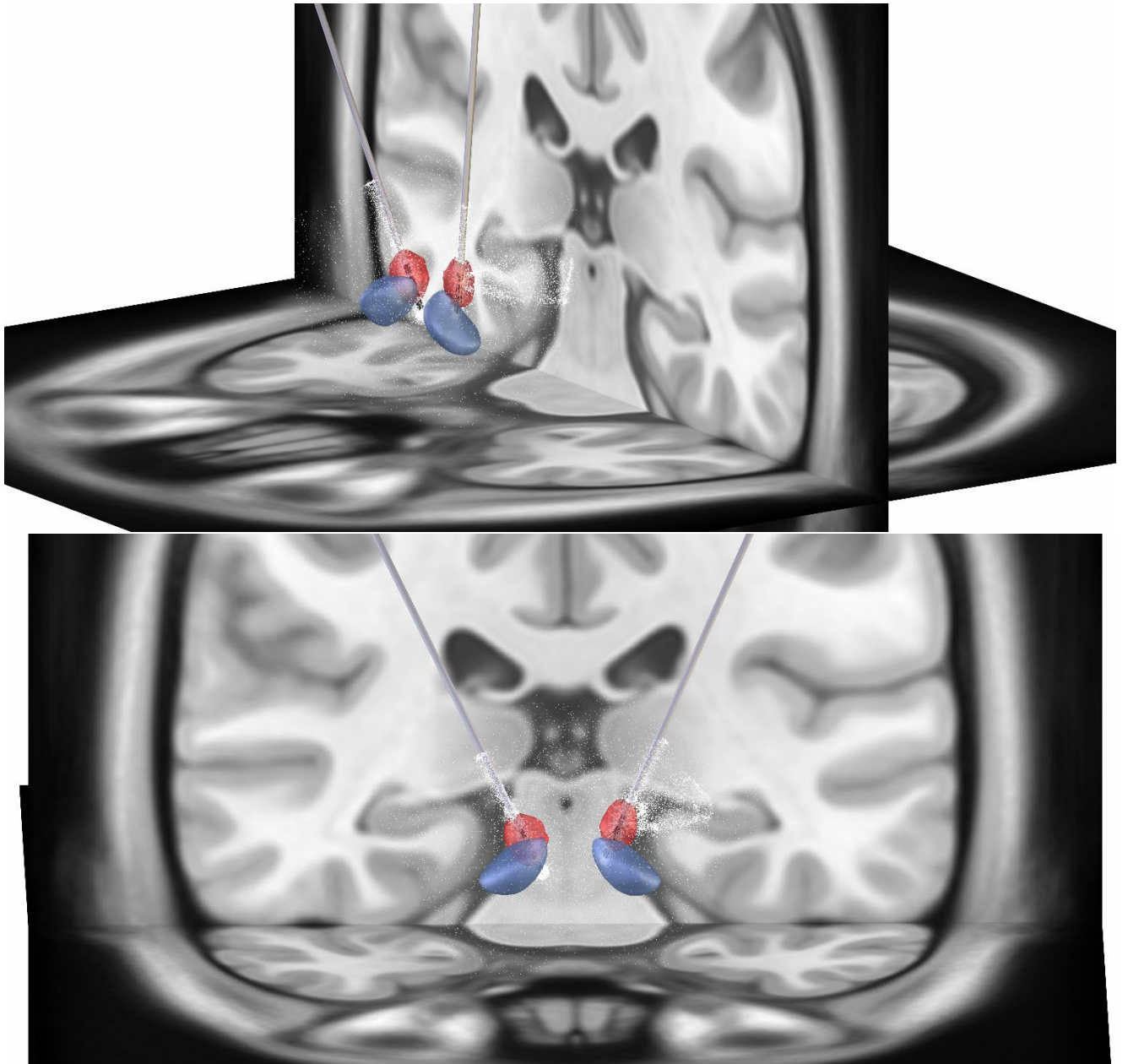

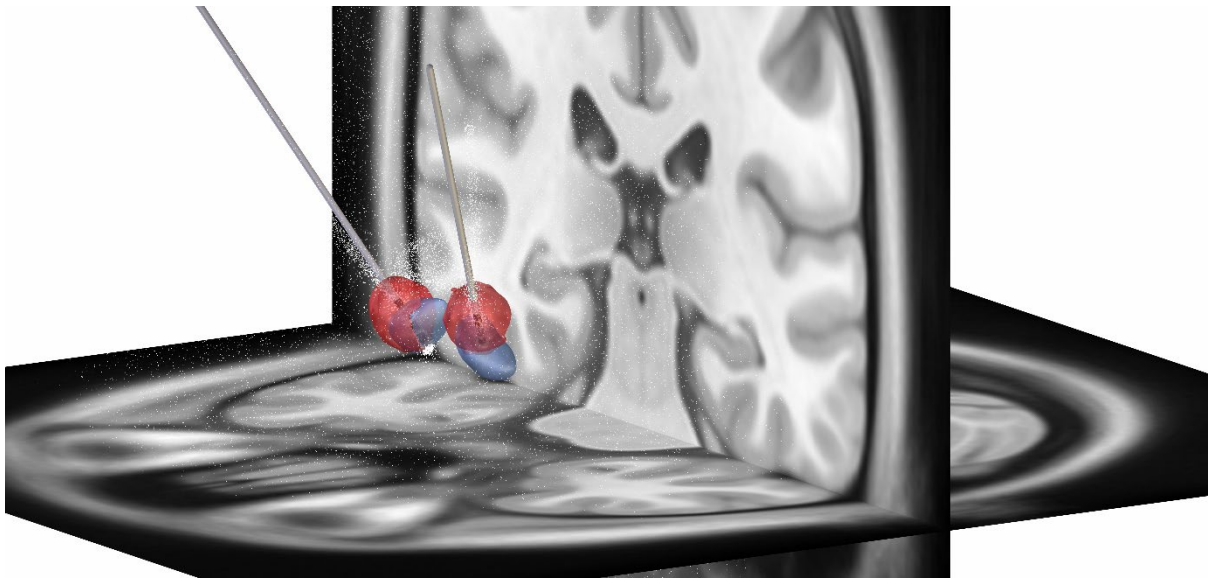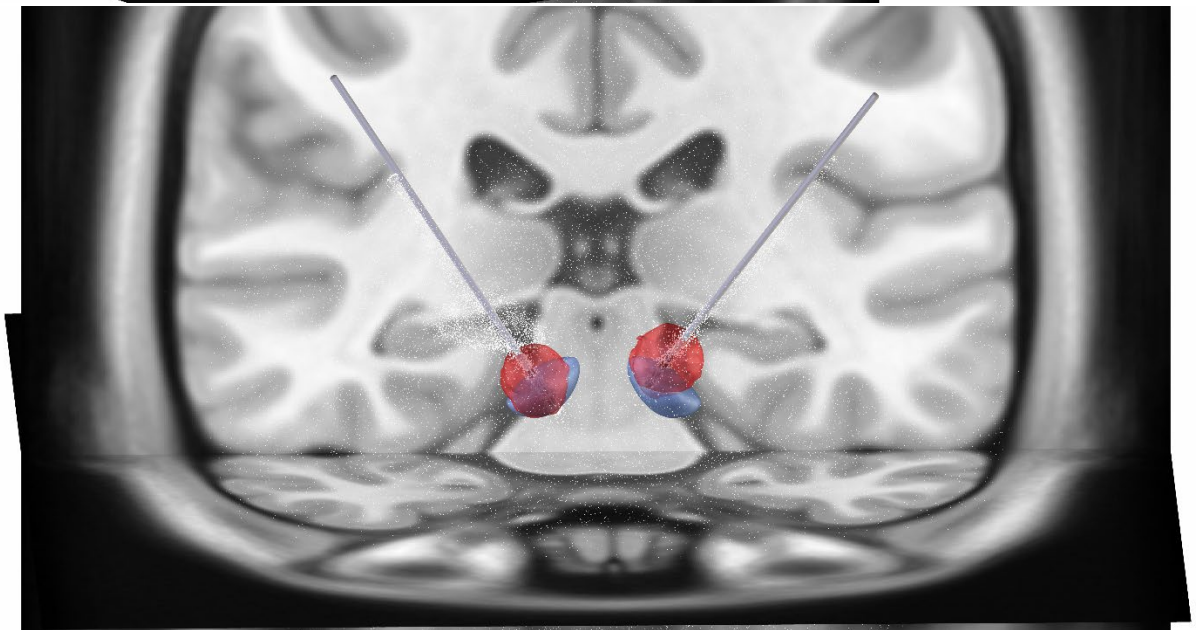

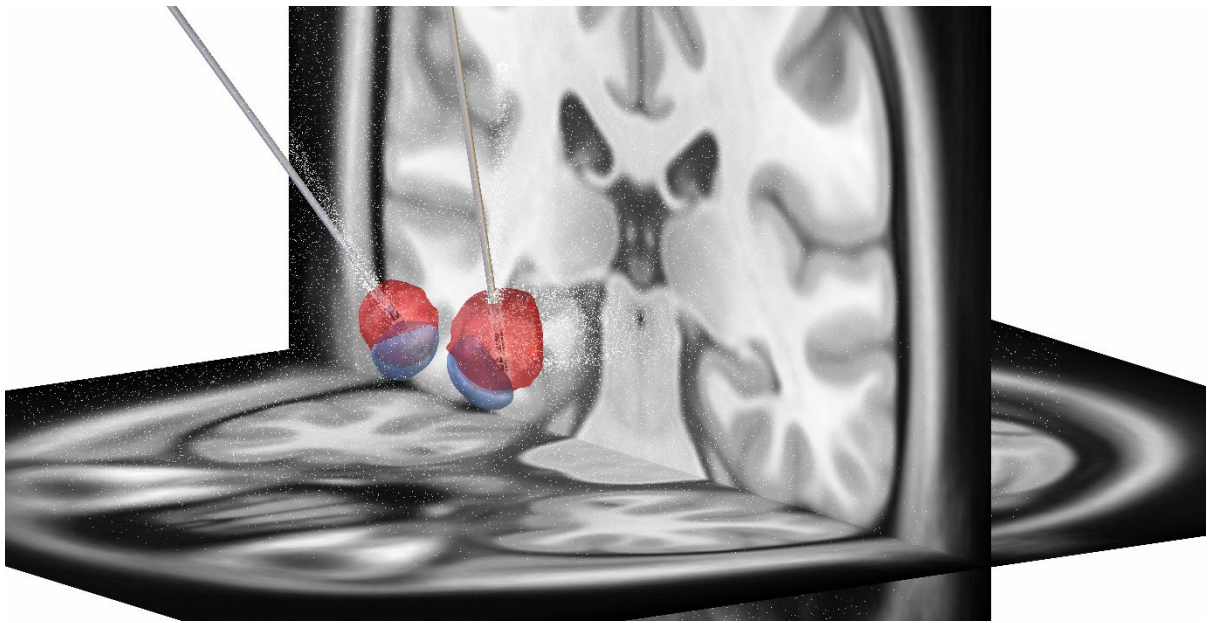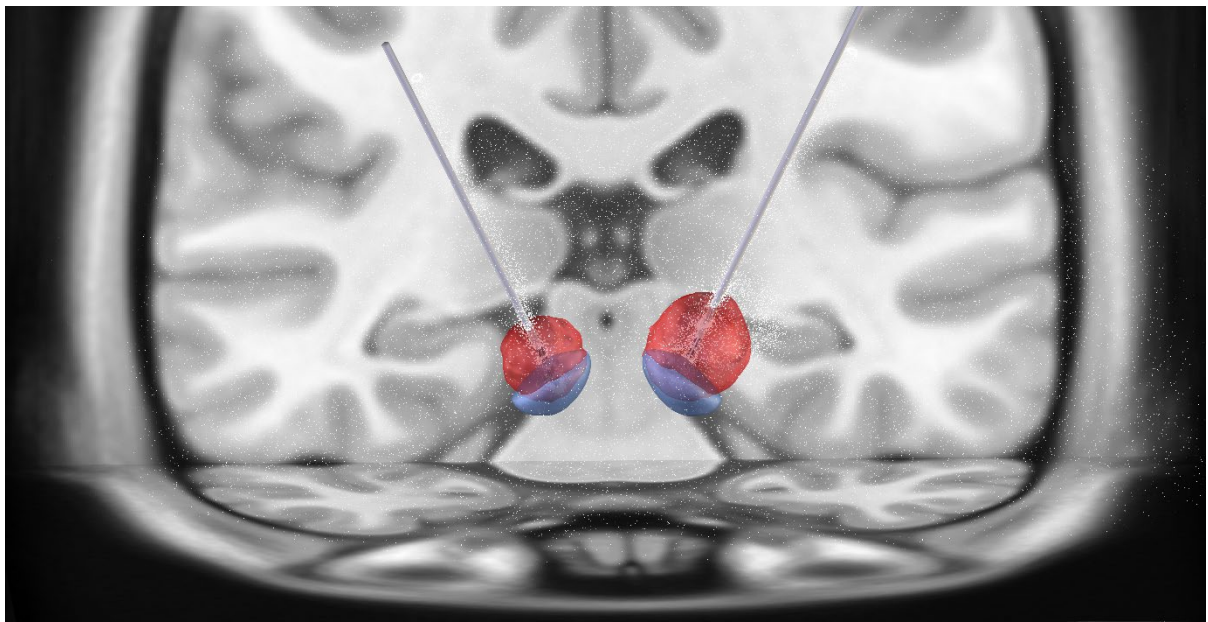

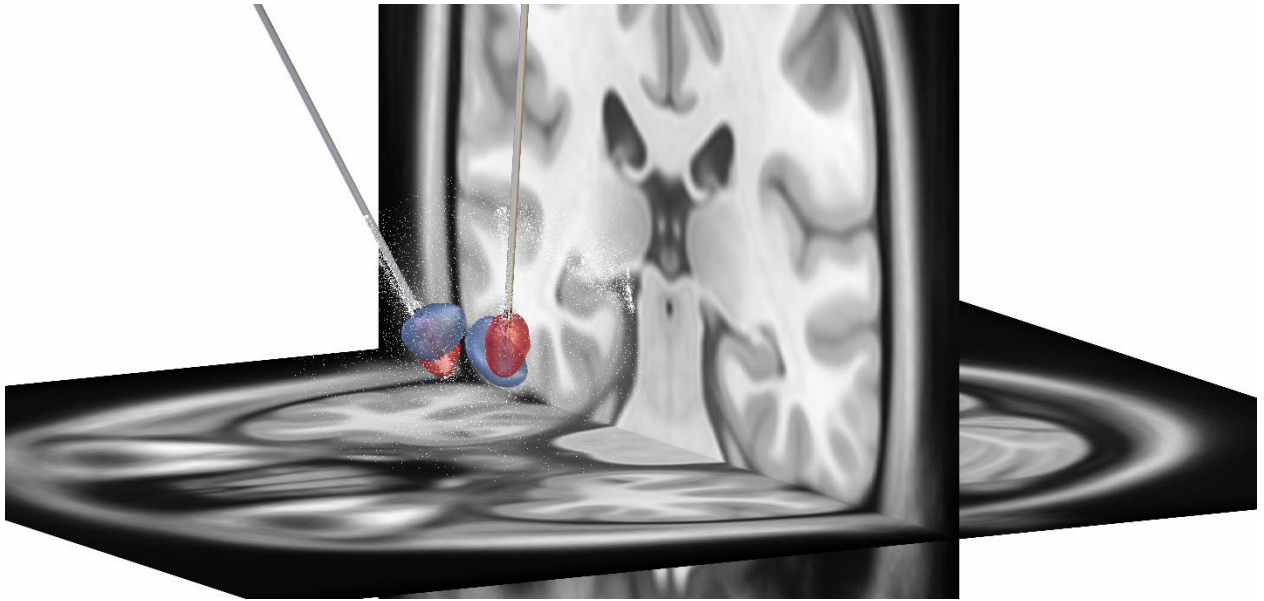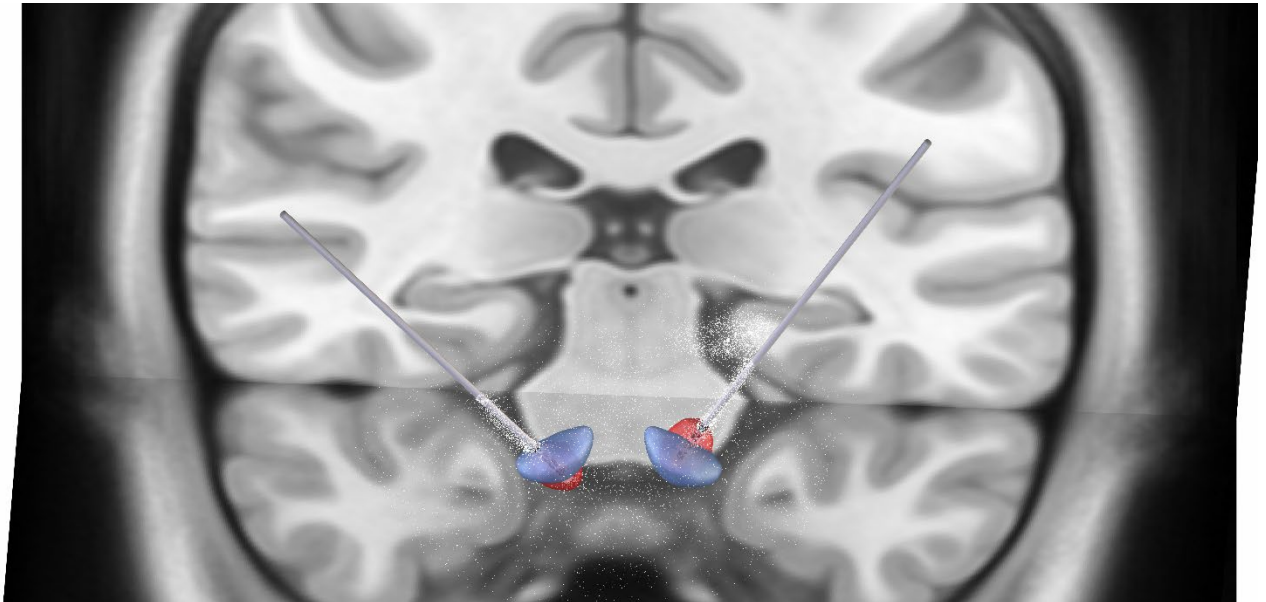

Supplement: Supplementary file 4 — Supplementary S4. Volume of tissue activated estimates according to final stimulation parameters for participants 1–8. [file PCN-78-131-s002.pdf]
